# SLC5A12 defines an invasive epithelial state associated with immune remodeling and therapeutic vulnerability in HNSCC

**DOI:** 10.3389/fcell.2026.1991399

**Published:** 2026-10-22

**Authors:** Yemei Tang, Huimin Zhang, Panpan Zhang, Huiwen Qu, Dandan Wu

**Affiliations:** 1 Department of Otorhinolaryngology, Suzhou Ninth People’s Hospital, Suzhou, China; 2 Shanxi University of Medicine, Fenyang, China

**Keywords:** ferroptosis, head and neck squamous cell carcinoma, molecular docking, single-cell RNA sequencing, SLC5A12, tumor microenvironment

## Abstract

**Background:**

Head and neck squamous cell carcinoma (HNSCC) is characterized by marked epithelial and immune heterogeneity. The malignant epithelial states associated with tumor progression, immune remodeling, and potential therapeutic vulnerability remain incompletely defined.

**Methods:**

Single-cell RNA-sequencing datasets from three HNSCC cohorts were integrated with TCGA-HNSC and five GEO cohorts. Epithelial-state heterogeneity, inferred trajectories, regulons, cell-cell communication, prognostic features, spatial localization, and ferroptosis-associated transcriptional programs were analyzed. SLC5A12 was prioritized through convergent computational analyses and was subsequently tested by loss-of-function assays and xenograft experiments. CellOracle perturbation, ferroptosis-related drug-sensitivity analyses, and molecular docking were treated as computational predictions.

**Results:**

Five epithelial states were identified, among which the Invasive population increased with disease progression and exhibited the highest inferred CNV burden. Invasive cells showed enrichment of malignant and extracellular-matrix programs and prominent predicted MIF-CD74/CXCR4 communication with regulatory T cells. Machine-learning, WGCNA, single-cell, and spatial analyses converged on SLC5A12 as an Invasive-state-associated prognostic feature. Computational analyses further indicated a ferroptosis-associated vulnerability profile in the Invasive state and predicted attenuation of invasive and immunoregulatory programs after virtual SLC5A12 loss. Experimentally, SLC5A12 knockdown reduced proliferation, clonogenicity, migration, invasion, and xenograft growth while increasing apoptosis. Structure-based virtual screening nominated ledipasvir (ZINC000150338819) and elbasvir (ZINC000150588351) as top-ranked candidate SLC5A12-binding compounds.

**Conclusion:**

SLC5A12 is a molecular feature of an Invasive epithelial state associated with HNSCC progression, adverse outcome, and predicted immune-remodeling and therapeutic-vulnerability programs. Experimental loss-of-function data support a tumor-promoting role for SLC5A12, whereas the MIF-Treg communication, ferroptosis vulnerability, CellOracle perturbation, and molecular-docking findings remain computational hypotheses requiring direct validation.

## 1 Introduction

Head and neck squamous cell carcinoma (HNSCC) is a biologically heterogeneous malignancy in which local invasion, nodal dissemination, recurrence, and resistance to therapy remain major determinants of outcome (Liu et al., 2019; Dai et al., 2020; Wu et al., 2020). Molecular studies have implicated multiple tumor-intrinsic processes in head and neck cancer progression, including non-coding RNA networks, oncogenic transcriptional programs, stromal metabolic adaptation, and regulated cell-death pathways (Prakash and Shaked, 2024; Duan et al., 2026; Shi et al., 2026). Together, these observations support the view that HNSCC progression reflects the coordinated behavior of distinct malignant programs rather than a single uniform epithelial phenotype.

The tumor microenvironment adds a second layer of heterogeneity. Immune, stromal, and malignant cells interact through cytokines, extracellular-matrix signals, and therapy-responsive pathways that influence immune escape and treatment efficacy in HNSCC (Li H. X. et al., 2024; Wang et al., 2021). Accordingly, the biological significance of a tumor-cell program is best interpreted together with the immune and stromal context in which that program occurs.

Single-cell and spatial transcriptomic approaches now permit direct resolution of this complexity. Studies of premalignant-to-malignant progression and established HNSCC have revealed stage-associated ecosystem remodeling, clinically relevant malignant epithelial states, specialized fibroblast populations, and spatially organized immune niches (Okuyama et al., 2023; Cao et al., 2024; Cai et al., 2023; Michaud et al., 2024; Wu et al., 2025). These findings provided the rationale for examining whether HNSCC progression is accompanied by expansion of a discrete epithelial state with coordinated invasive and immunoregulatory features.

Among candidate mediators of tumor-immune communication, macrophage migration inhibitory factor (MIF) is a pleiotropic cytokine involved in inflammatory signaling and immune-cell regulation and can act through receptor complexes involving CD74 and chemokine receptors (Ruiz-Torres et al., 2025; Sumaiya et al., 2022; Bozza et al., 2020 He et al., 2021). We therefore examined whether MIF-associated signaling was preferentially linked to a defined malignant epithelial population and whether the same population was associated with regulatory T-cell features.

SLC5A12 encodes a sodium-coupled monocarboxylate transporter. Recent studies have connected SLC5A12 to lactate-dependent inflammatory signaling and to tumor-associated metabolic and prognostic phenotypes (Certo et al., 2025; Lin et al., 2025). Its cellular localization and functional relevance in HNSCC, however, remain poorly defined. Here, we integrated three HNSCC single-cell datasets with TCGA-HNSC, five independent GEO cohorts, spatial transcriptomics, network analysis, machine-learning survival modeling, ferroptosis-related computational analyses, *in silico* perturbation, loss-of-function experiments, xenograft validation, and structure-based virtual screening. This framework was used to define an Invasive epithelial state, determine the position of SLC5A12 within that state, and evaluate its associations with immune remodeling, clinical outcome, and predicted therapeutic vulnerability.

## 2 Materials and methods

### 2.1 Acquisition and preprocessing of single-cell RNA-seq data

Single-cell RNA sequencing (scRNA-seq) datasets of HNSCC were retrieved from the Gene Expression Omnibus (GEO), including GSE181919, GSE182227, and GSE234933. The downloaded expression matrices contained 54,239, 70,970, and 187,399 cells, respectively (312,608 cells in total). For GSE234933, the analysis started from called cells defined in the annotation table, with empty droplets excluded; the downloaded GSE182227 barcode file contained 70,970 cell barcodes. Genes expressed in fewer than four cells were removed before cell-level quality control. Cells were retained when they contained 200–5,000 detected genes, 500–25,000 total UMI counts, a mitochondrial transcript proportion <10%, and a hemoglobin-transcript proportion <5%. After this additional quality-control procedure, 49,003 cells from GSE181919, 63,927 cells from GSE182227, and 171,214 cells from GSE234933 were retained, yielding 284,144 cells for downstream analysis.

After quality filtering, retained cells were normalized using Seurat LogNormalize and scaled for downstream analysis. Highly variable features were selected with the variance-stabilizing transformation method, and the top 2,000 genes were retained. Principal component analysis (PCA) was performed, and the first 30 principal components were supplied to Harmony for batch correction across datasets. The first 30 Harmony dimensions were then used for shared-nearest-neighbor construction and UMAP visualization.

Cellular clusters were generated from the Harmony-corrected shared-nearest-neighbor graph using Seurat FindNeighbors and FindClusters. The global clustering resolution was 0.5. Major cell populations were annotated using established lineage markers, including EPCAM/KRT-family genes for epithelial cells, CD3D for T cells, COL1A1/DCN for fibroblasts, and canonical endothelial, myeloid, B-cell, and plasma-cell markers shown in Figure 1B. Epithelial cells were subsequently extracted and reclustered using the first 30 Harmony dimensions at a resolution of 1.0.

**FIGURE 1:**
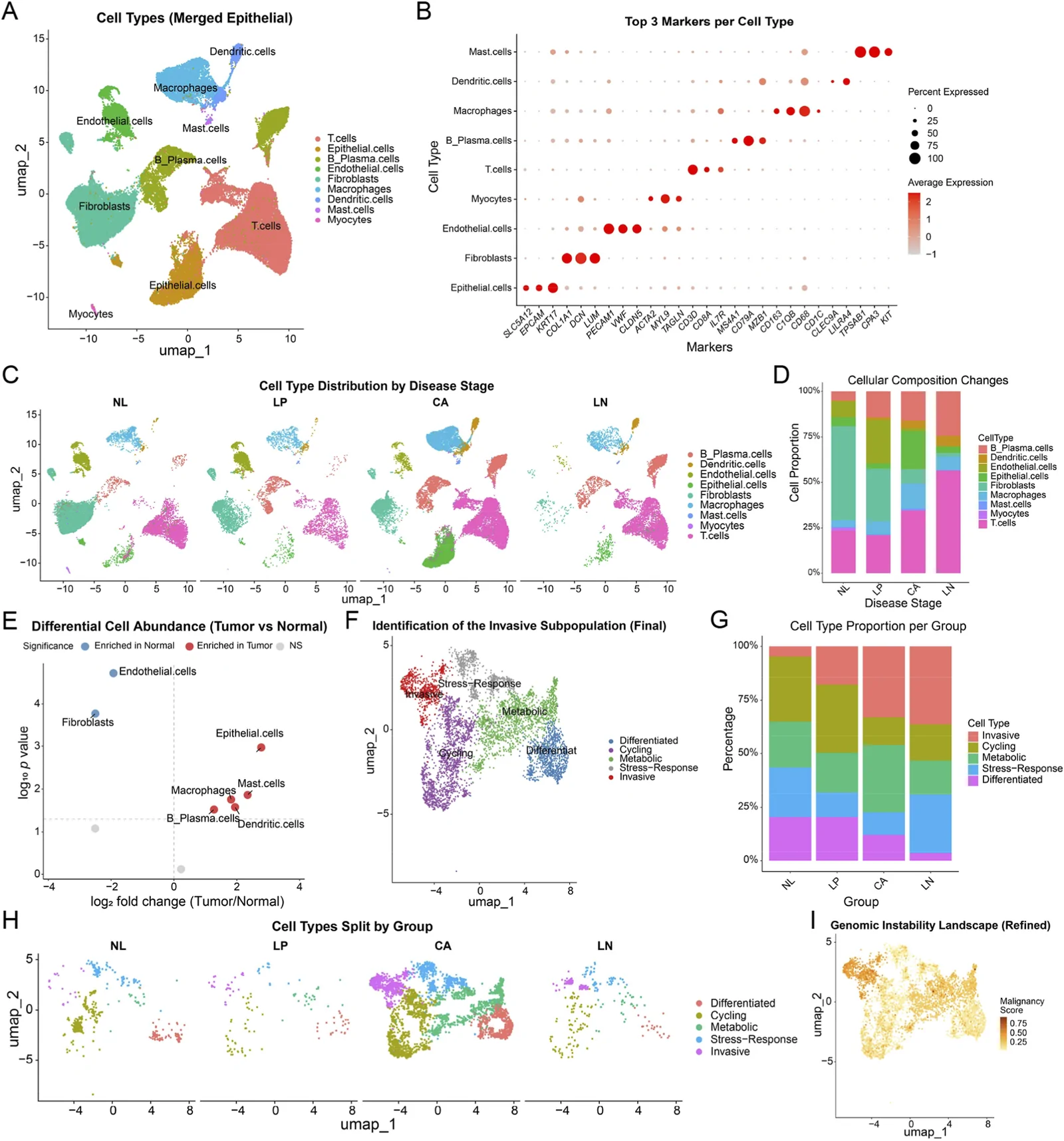
Single-cell mapping delineates cellular ecosystem remodeling and an Invasive epithelial state across HNSCC progression. **(A)** Integrated UMAP of the major cell populations identified after quality control and dataset integration. Major compartments include epithelial, T/NK, macrophage, fibroblast, endothelial, and additional immune/stromal populations. **(B)** Dot/bubble plot of canonical lineage markers used to support cell-type annotation; dot size and color represent the quantities indicated in the panel. **(C)** UMAP split by pathological stage: normal mucosa (NL), leukoplakia (LP), primary carcinoma (CA), and lymph-node metastasis (LN). **(D)** Relative composition of major cell populations across the four stages. **(E)** Differential-abundance comparison showing stage-associated changes in cellular composition. **(F)** Secondary clustering of epithelial cells into five transcriptional states: Invasive, Metabolic, Cycling, Stress-Response, and Differentiated. **(G)** Relative abundance of the five epithelial states across pathological stages. **(H)** Stage-resolved UMAP distribution of epithelial states. **(I)** inferCNV analysis using T cells as the non-malignant reference; the heatmap and quantitative score summarize inferred large-scale copy-number deviations, with the Invasive population showing the greatest overall CNV deviation. Statistical significance is displayed in the corresponding panels; all tests were two-sided as described in Methods.

### 2.2 Inference of copy number variations (CNV) and malignancy scoring

To compare malignant-associated transcriptional CNV patterns among epithelial populations, large-scale copy-number variation (CNV) profiles were inferred using the inferCNV R package. Annotated T-cell populations were used as a putative non-malignant diploid reference. Expression values were ordered by genomic position and smoothed to identify relative deviations from the reference profile. Because inferCNV estimates CNV-like patterns from transcript abundance, these profiles were interpreted as inferred transcription-based CNV deviations rather than direct genomic measurements.

For individual epithelial cells, an inferred CNV score was derived by quantifying the overall deviation of the inferCNV profile from the reference state. This score was used to compare relative CNV-like transcriptional burden among epithelial subpopulations and was not interpreted as a direct measure of genomic instability.

### 2.3 Epithelial cell sub-clustering and trajectory analysis

Epithelial cells were isolated from the integrated single-cell dataset and subjected to secondary clustering. State labels were assigned by jointly considering cluster-specific marker expression and functional enrichment. The Invasive state was characterized by SLC5A12, MMP1, MIF, and invasion/ECM-associated programs; Cycling cells by proliferation markers including MKI67, TOP2A, and CCNB1; Differentiated cells by terminal epithelial markers including SPRR3, CNFN, KRT1, and IVL; and Stress-Response cells by stress-adaptive markers including NDRG1, SLC2A1, FABP5, and EGR1. The Metabolic state was defined primarily by its coordinated enrichment of metabolic transcriptional programs rather than by a single dominant marker gene. These complementary marker- and pathway-based criteria resolved five epithelial states: Invasive, Metabolic, Cycling, Stress-Response, and Differentiated.

To investigate transcriptional relationships among epithelial states, trajectory reconstruction was performed using Monocle 2. Ordering genes were not manually preselected; instead, all genes meeting q < 0.01 in differentialGeneTest with epithelial subcluster identity as the explanatory variable were passed to setOrderingFilter. Dimensionality reduction was then performed with DDRTree using two components, followed by pseudotime ordering with orderCells. The branch enriched for Differentiated cells and terminal differentiation markers, including KRT1, IVL, SPRR3, and CNFN, was designated as the root, whereas increasing SLC5A12, MIF, and MMP1 expression characterized the Invasive-directed region. Thus, trajectory orientation was anchored by reciprocal differentiation- and invasion-associated transcriptional features rather than by chronological information. Pseudotime was interpreted strictly as an inferred transcriptional continuum and not as direct evidence of lineage ancestry or temporal progression.

Pseudotime was interpreted strictly as an inferred transcriptional continuum. It was not used as evidence of lineage ancestry, temporal progression, or a deterministic Differentiated-to-Invasive developmental path.

### 2.4 Regulatory network inference

To identify transcriptional regulatory programs associated with the invasive epithelial phenotype, SCENIC-based regulon analysis was conducted using the SCENIC R workflow. Gene regulatory networks were reconstructed by integrating transcription factor expression patterns with co-expressed candidate target genes. Subsequently, regulon specificity was determined through transcription factor binding motif enrichment analysis, and regulon activity scores were calculated using the AUCell algorithm.

Transcription factors exhibiting preferentially enhanced regulon activity in the invasive epithelial population were selected as candidate regulators involved in shaping the invasive transcriptional state. Regulatory factors including FOSL1, STAT3, RELA, and TEAD1 were considered potential upstream modulators based on computationally inferred regulon activation patterns.

### 2.5 Cell-cell communication analysis

To characterize potential intercellular signaling networks within the HNSCC tumor microenvironment, cell–cell communication inference was performed using the CellChat R package based on normalized single-cell transcriptomic profiles. The CellChatDB human ligand–receptor interaction database was applied to evaluate putative signaling events involving secreted factors, extracellular matrix-associated interactions, and direct cell-contact communication.

Significant ligand–receptor interactions were identified according to estimated communication probabilities generated by the CellChat algorithm. Analyses were further focused on signaling interactions involving the invasive epithelial population and immune cell subsets, with particular emphasis on the MIF signaling pathway and its predicted relationship with regulatory T-cell-associated communication networks.

All CellChat-derived interactions were considered computational predictions of potential intercellular signaling patterns and were not interpreted as direct experimental evidence of ligand–receptor activation.

### 2.6 Bulk transcriptome profiling and weighted gene co-expression network analysis (WGCNA)

Bulk transcriptomic profiles of HNSCC samples together with matched clinical annotations were obtained from The Cancer Genome Atlas (TCGA) via the UCSC Xena platform. The expression matrices were subjected to standard preprocessing procedures before downstream co-expression and survival-related analyses.

Weighted gene co-expression network analysis (WGCNA) was subsequently applied to construct co-expression modules associated with tumor invasive characteristics and immune-related features. The soft-thresholding parameter was determined according to the scale-free topology criterion, with a goodness-of-fit index exceeding 0.9. Module eigengenes (MEs) were then calculated and correlated with relevant clinical and immune-associated traits to identify biologically meaningful modules.

An Invasive epithelial signature score was generated by ssGSEA using marker genes defining the Invasive population identified from scRNA-seq analysis. Treg abundance was estimated using CIBERSORT-derived infiltration scores. Module eigengenes were correlated with these two quantitative traits. The Green module showed the strongest positive relationship with both traits and was selected for candidate prioritization. SLC5A12 was highlighted because it combined high module membership and high gene significance within the Green module and was independently supported by single-cell localization, survival analysis, spatial analysis, and multiple feature-selection approaches. No arbitrary hard cutoff for module membership or gene significance was used to define SLC5A12 as a hub; prioritization was based on joint high values and cross-analysis convergence.

### 2.7 Survival analysis and machine learning-based predictive modeling

To establish a prognostic model reflecting invasive epithelial-associated molecular characteristics, survival analyses and machine-learning-based modeling were performed using the TCGA-HNSCC cohort for model development and independent GEO cohorts for external validation.

Initially, genes significantly associated with overall survival were screened using univariate Cox proportional hazards regression. Variables passing initial screening were further assessed through multivariate Cox regression analysis to determine their independent prognostic relevance.

An integrated survival modeling framework was subsequently developed by incorporating multiple machine-learning algorithms, including Least Absolute Shrinkage and Selection Operator (LASSO) Cox regression, Ridge Cox regression, Stepwise Cox regression, Support Vector Machine for Survival (survival-SVM), Gradient Boosting Machine (GBM), Random Survival Forest (RSF), and Cox Boost regression.

More than 100 algorithmic combinations and parameter configurations were evaluated, with performance assessed by the concordance index (C-index) in TCGA-HNSC and five independent GEO cohorts. Cross-cohort evaluation, rather than training-set performance alone, was used to reduce model-selection bias. Among the displayed top-ranked configurations, PLSR + CoxBoost showed the most balanced cross-cohort performance and was selected for the revised prognostic framework. Its C-index values were 0.689 in GSE171973, 0.690 in GSE39366, 0.716 in GSE41613, 0.711 in GSE42743, 0.774 in GSE65858, and 0.784 in TCGA-HNSC.

For each patient, the prognostic risk score was calculated as the sum of each selected gene-expression value multiplied by its fitted model coefficient. The TCGA-HNSC training cohort was dichotomized using its median risk score. For each of the five independent GEO validation cohorts, the median risk score was calculated within that cohort and used as the cohort-specific cutoff; patients above the median were assigned to the high-risk group and those at or below the median to the low-risk group. Kaplan-Meier curves and log-rank tests were used to compare overall survival. Cross-cohort evaluation in five independent GEO datasets was used to reduce dependence on training-cohort performance and to assess model generalizability.

### 2.8 Cell culture and generation of stable cell lines

Human HNSCC cell lines FaDu and CAL27 were purchased from Procell Biotechnology (China). Cells were maintained in RPMI-1640 medium containing 10% fetal bovine serum (FBS) and 1% penicillin–streptomycin and incubated at 37 °C in a humidified atmosphere supplemented with 5% CO_2_. Cell identity was verified by short tandem repeat (STR) analysis.

Lentiviral constructs carrying SLC5A12-targeting short hairpin RNAs (sh-SLC5A12) or a corresponding non-targeting control sequence (sh-NC) were provided by Genechem Co., Ltd (Shanghai, China). The targeting sequences were as follows:

sh-SLC5A12#1: 5′-CTG​TAC​CCT​GGG​AGG​ATT​AAA-3′

sh-SLC5A12#2: 5′-GAT​ATT​CAA​CCA​CTG​TTA​ATT-3′

For lentiviral transduction, FaDu and CAL27 cells were infected at approximately 20%–30% confluence. Stable knockdown populations were established through puromycin selection (1 μg/mL) for 7 days beginning 72 h after infection. The efficiency of SLC5A12 silencing was subsequently assessed by Western blot analysis.

### 2.9 Western blotting

Western blotting was performed according to previously described protocols (Liu et al., 2019). Briefly, extracted proteins were resolved by SDS-PAGE and subsequently transferred onto membrane substrates. The membranes were incubated with primary antibodies targeting SLC5A12 (Santa Cruz Biotechnology, sc-515141) and β-actin (Proteintech, 66009-1-Ig; 1:10,000), followed by treatment with corresponding HRP-linked secondary antibodies. Protein expression signals were visualized using conventional chemiluminescence-based detection methods.

### 2.10 Assessment of cell proliferation

Cell growth capacity was examined using complementary CCK-8 viability assays and colony formation assays. For CCK-8 analysis, stable FaDu and CAL27 cell populations were plated into 96-well culture plates. At predefined intervals (0, 24, 48, 72, and 96 h), 10 μL of CCK-8 solution was added to each well, followed by incubation in darkness for 2 h. Absorbance measurements were obtained according to the manufacturer’s recommended protocol. All experimental groups were assessed using three technical replicates.

For clonogenic assays, cells were distributed into six-well plates and maintained in culture for 14 days. The medium was renewed every 7 days throughout the incubation period. Following colony development, cells were fixed with 4% paraformaldehyde, stained with 0.5% crystal violet, and colonies were manually enumerated. Three independent biological experiments were conducted for each condition.

### 2.11 Migration and invasion analysis

The migratory and invasive capacities of HNSCC cells were assessed using Transwell-based assays. For migration experiments, cells were introduced into the upper compartments of Transwell inserts without extracellular matrix coating. For invasion assays, the upper surfaces of the inserts were coated with Matrigel prior to cell loading. Cells were resuspended in serum-free medium and placed in the upper chambers, while the lower compartments were filled with medium containing 20% FBS to provide a chemotactic stimulus.

Following incubation, cells that had traversed the membrane were fixed with 0.5% crystal violet staining solution and subsequently quantified by microscopic enumeration. At least three randomly selected microscopic fields were evaluated for each insert. All experiments were independently performed in triplicate.

### 2.12 *In vivo* subcutaneous xenograft model

All animal procedures were reviewed and approved by the Ethics Committee for Laboratory Animal Welfare of Shanxi University of Medicine (approval number: 2025041). Female BALB/c nude mice (4 weeks of age; five animals per group) were housed in a specific pathogen-free environment with unrestricted access to food and water.

FaDu cells with stable SLC5A12 knockdown (sh-SLC5A12#2) or control transduction (sh-NC) were subcutaneously implanted into the bilateral axillary regions of recipient mice. Tumor growth was monitored every 2 days starting from day 7 after inoculation, and tumor volumes were determined according to the formula: Tumor volume = length × width^2^/2.

At day 25 following cell implantation, mice were euthanized by CO_2_ inhalation, followed by cervical dislocation to ensure death. Excised tumors were photographed, collected, and weighed, and tumor specimens were subsequently subjected to histological and immunohistochemical evaluation. All procedures were conducted in compliance with institutional regulations governing laboratory animal care.

### 2.13 In silico perturbation analysis of SLC5A12 at single-cell resolution

To explore the potential transcriptional consequences associated with SLC5A12 depletion at single-cell resolution, a computational perturbation framework was applied using Cell Oracle based on the reconstructed gene regulatory network (GRN) of the invasive epithelial population.

Single-cell transcriptomic profiles from SLC5A12-expressing invasive epithelial cells were extracted, and a cell-state-specific regulatory network was generated following the Cell Oracle workflow. Virtual suppression of SLC5A12 was introduced into the inferred network model to simulate gene perturbation, and subsequent changes in downstream transcriptional states were computationally estimated.

The predicted impact of SLC5A12 perturbation was assessed by comparing simulated control and perturbed cellular states. Altered genes, associated pathway activities, and transcriptional signatures reflecting epithelial-state changes were subsequently evaluated to characterize the predicted regulatory effects.

The *in silico* knockout analysis was considered a computational prediction of potential consequences following SLC5A12 loss and was not interpreted as an experimental replacement for genetic deletion approaches.

### 2.14 Spatial transcriptomic analysis

Spatial transcriptomic analysis was performed using two 10x Genomics Visium HNSCC sections from GSE181300: GSM5494475 (HNSCC201125T04; HNSC1) and GSM5494476 (HNSCC201125T05; HNSC2). The corresponding Seurat objects were loaded and analyzed using the Cottrazm (Construction of Tumor Transition Zone Microenvironment) spatial deconvolution framework. Cottrazm resolves the tumor spatial microenvironment through three linked procedures. First, Cottrazm-BoundaryDefine integrates spatial transcriptomic profiles with H&E-stained histological images to identify malignant, boundary, and non-malignant spatial regions. Second, Cottrazm-SpatialDecon integrates the spatial transcriptomic data, the reference single-cell transcriptomic data, and spot coordinates to infer the cellular composition of each Visium spot. Third, Cottrazm-SpatialRecon reconstructs cell-type-specific gene-expression profiles at sub-spot resolution. This workflow follows the Cottrazm framework described by Xun et al. (2023) and the spatial analytical strategy used in the SLC41A3 study (Gong et al., 2026).

For the analyses presented here, spots were grouped according to the deconvolved TumorCells proportion: spots with TumorCells >0 were classified as malignant (Mal), whereas spots with TumorCells = 0 were classified as non-malignant (nMal). SLC5A12 expression between Mal and nMal spots was compared separately within each section using the Wilcoxon rank-sum test. To characterize the spatial relationship between SLC5A12 and the cellular composition of the local microenvironment, Spearman correlation analysis was performed between SLC5A12 expression and Cottrazm-SpatialDecon-estimated cell-type proportions across spatial spots. Spatial associations were interpreted as localization and co-variation patterns rather than evidence of causal regulation between cell populations.

### 2.15 Ferroptosis-associated computational analysis

Ferroptosis-associated transcriptional features were evaluated using a literature-informed panel of ten representative regulators selected to cover three established functional dimensions of ferroptosis: polyunsaturated-lipid oxidation, iron handling/ferritinophagy, and glutathione-GPX4 antioxidant protection. The panel comprised ACSL4, LPCAT3, ALOX15, TFRC, NCOA4, GPX4, SLC7A11, FTH1, GCLC, and GCLM and was assembled as a representative functional set rather than downloaded as a single predefined database signature. Five module scores were derived from this panel: an overall ferroptosis score based on all ten genes; a ferroptosis-driver score based on ACSL4, LPCAT3, ALOX15, TFRC, and NCOA4; a ferroptosis-suppressor score based on GPX4, SLC7A11, FTH1, GCLC, and GCLM; a lipid-peroxidation score based on ACSL4, LPCAT3, and ALOX15; and an iron-metabolism score based on TFRC, NCOA4, and FTH1. Module scores were calculated from log-normalized single-cell expression using Seurat AddModuleScore with 24 expression bins and 100 expression-matched control genes per input gene.

KEGG-based gene set enrichment analysis was performed with clusterProfiler gseKEGG using human pathways (organism = “hsa”). Genes with valid mapped identifiers were ranked in descending order of log2 fold change. The fgsea backend was used with an enrichment exponent of 1 and gene-set sizes of 10–500 genes. *P* values were adjusted using the Benjamini-Hochberg method, with adjusted *P* < 0.05 considered significantly enriched.

Predicted drug-response patterns were characterized using Beyondcell drug-signature scoring to prioritize candidate compounds across epithelial subpopulations, including erastin, RSL3, and ML210. FI-X was analyzed separately as a hypothetical mechanism-based ferroptosis-inducer score. The ferroptosis-promoting module comprised ACSL4, LPCAT3, ALOX15, TFRC, and NCOA4, whereas the protective module comprised GPX4, SLC7A11, FTH1, GCLC, and GCLM. The FI-X raw score was calculated as the promoting-module score minus the protective-module score and was subsequently min-max scaled to 0–1 across pooled epithelial cells. Higher values represent a transcriptional balance predicted to favor susceptibility to the hypothetical ferroptosis-inducing perturbation. All ferroptosis and drug-sensitivity results were treated as computational vulnerability estimates rather than experimental evidence of ferroptotic cell death.

### 2.16 Virtual screening and molecular docking

Potential SLC5A12-targeting compounds were identified by virtual screening of FDA-approved drugs. Ligand structures were obtained from the ZINC database, the SLC5A12 protein sequence from UniProt, and the three-dimensional SLC5A12 structure from AlphaFold. Protein preparation, including removal of water molecules and addition of polar hydrogens, and ligand preparation, including charge assignment and definition of rotatable bonds, were performed using AutoDockTools v1.2.7. Initial screening was conducted with AutoDock Vina v1.2.7 using exhaustiveness = 4. The ten compounds with the most favorable predicted docking energies were then subjected to refined docking using a search box centered at x = 2.031, y = 4.912, and z = −11.067, with box dimensions x = 29.521986928783832, y = 24.503641209149926, and z = 37.32394628977717. Refined docking used exhaustiveness = 32, seed = 42, num_modes = 10, and energy_range = 3 kcal/mol. The lowest-energy pose was retained for structural inspection. Three-dimensional complexes were visualized using PyMOL v3.1.0, and two-dimensional interaction diagrams were generated using Discovery Studio v2019. AutoDock Vina values are reported as predicted docking energies/scores rather than experimentally measured binding free energies. ZINC000150338819 corresponds to ledipasvir and ZINC000150588351 to elbasvir.

### 2.17 Statistical analysis

Statistical analyses were conducted using R software (version 3.6.0) unless otherwise indicated. Continuous variables were analyzed using Student’s t-test or the Wilcoxon rank-sum test according to the distributional characteristics and data type of the comparison. For the spatial transcriptomic analysis, Mal-versus-nMal comparisons were performed separately within each HNSC section using the Wilcoxon rank-sum test, and SLC5A12-cell-abundance relationships were assessed using Spearman correlation. For spatial group summaries, the mean, standard error, and number of spots were reported. Formal standalone normality and variance-homogeneity tests were not separately documented in the original analysis workflow; accordingly, the revised manuscript does not retrospectively assign such tests. Transcriptome-wide multiple comparisons were controlled using the Benjamini-Hochberg false-discovery rate when applicable. Survival outcomes were evaluated using Kaplan-Meier analysis with log-rank testing, and Cox proportional-hazards models were applied where indicated. All statistical tests were two-sided, and P < 0.05 was considered statistically significant.

For *in vitro* experiments, the numbers of biological and technical replicates are specified in the corresponding Methods and figure legends, and data are presented as mean ± SD where indicated. For the xenograft study, the mouse was treated as the independent biological unit (n = 5 mice per group); measurements from bilateral tumors within the same mouse were averaged before statistical analysis. No animal was excluded from the final analysis.

## 3 Results

### 3.1 Single-cell profiling reveals progressive remodeling of the HNSCC ecosystem and resolves an invasive epithelial state

To define how the cellular architecture of HNSCC changes across disease progression, we integrated publicly available single-cell RNA-sequencing datasets spanning normal mucosa (NL), leukoplakia (LP), primary carcinoma (CA), and lymph-node metastasis (LN). Unsupervised clustering and UMAP visualization resolved nine major cellular compartments, including epithelial, T-cell, macrophage, fibroblast, endothelial, and other immune and stromal populations, which were annotated using canonical lineage markers (Figures 1A,B). The resulting atlas provided a common cellular framework in which epithelial and immune remodeling could be examined across pathological stages.

Comparison of cell-type composition revealed a marked redistribution of the tumor ecosystem during malignant progression. Epithelial populations expanded from NL and LP toward CA and LN, whereas several stromal populations, including fibroblasts and endothelial cells, were relatively reduced in malignant tissues (Figures 1C–E). Immune-cell composition also shifted across stages, indicating that epithelial expansion occurred in parallel with broader microenvironmental remodeling rather than as an isolated change in tumor-cell abundance.

We next isolated epithelial cells and performed secondary clustering to resolve malignant-state heterogeneity. Five transcriptionally distinct epithelial states were identified: Invasive, Metabolic, Cycling, Stress-Response, and Differentiated (Figure 1F). These populations occupied discrete regions of the UMAP space, with Invasive and Metabolic cells clearly separated from Differentiated and Stress-Response cells, indicating substantial transcriptional diversification within the epithelial compartment.

The distribution of these states changed systematically with disease stage. Differentiated and Stress-Response cells predominated in non-malignant and premalignant tissues, whereas Invasive and Metabolic populations became increasingly represented in carcinoma and metastatic samples (Figures 1G,H). In particular, the Invasive population accumulated in CA and LN tissues, linking this state to malignant progression. InferCNV analysis provided an independent measure of malignant-associated genomic deviation: among the five epithelial states, Invasive cells displayed the highest inferred CNV score (Figure 1I). Thus, stage-dependent expansion, transcriptional identity, and inferred copy-number alterations converged on the Invasive population as the epithelial state most closely associated with advanced HNSCC.

### 3.2 The Invasive epithelial state is defined by dedifferentiation, matrix-remodeling programs, and coordinated regulon activity

To determine the molecular features that distinguish Invasive cells from other epithelial states, we compared state-specific transcriptional profiles. Invasive cells showed increased expression of genes linked to tissue invasion and microenvironmental interaction, including MMP1, CXCL14, and MIF, together with reduced expression of terminal epithelial differentiation markers such as KRT1 and IVL (Figure 2A). This combination of invasion-associated gene induction and loss of differentiation markers defined the Invasive cluster as a comparatively dedifferentiated epithelial state.

**FIGURE 2:**
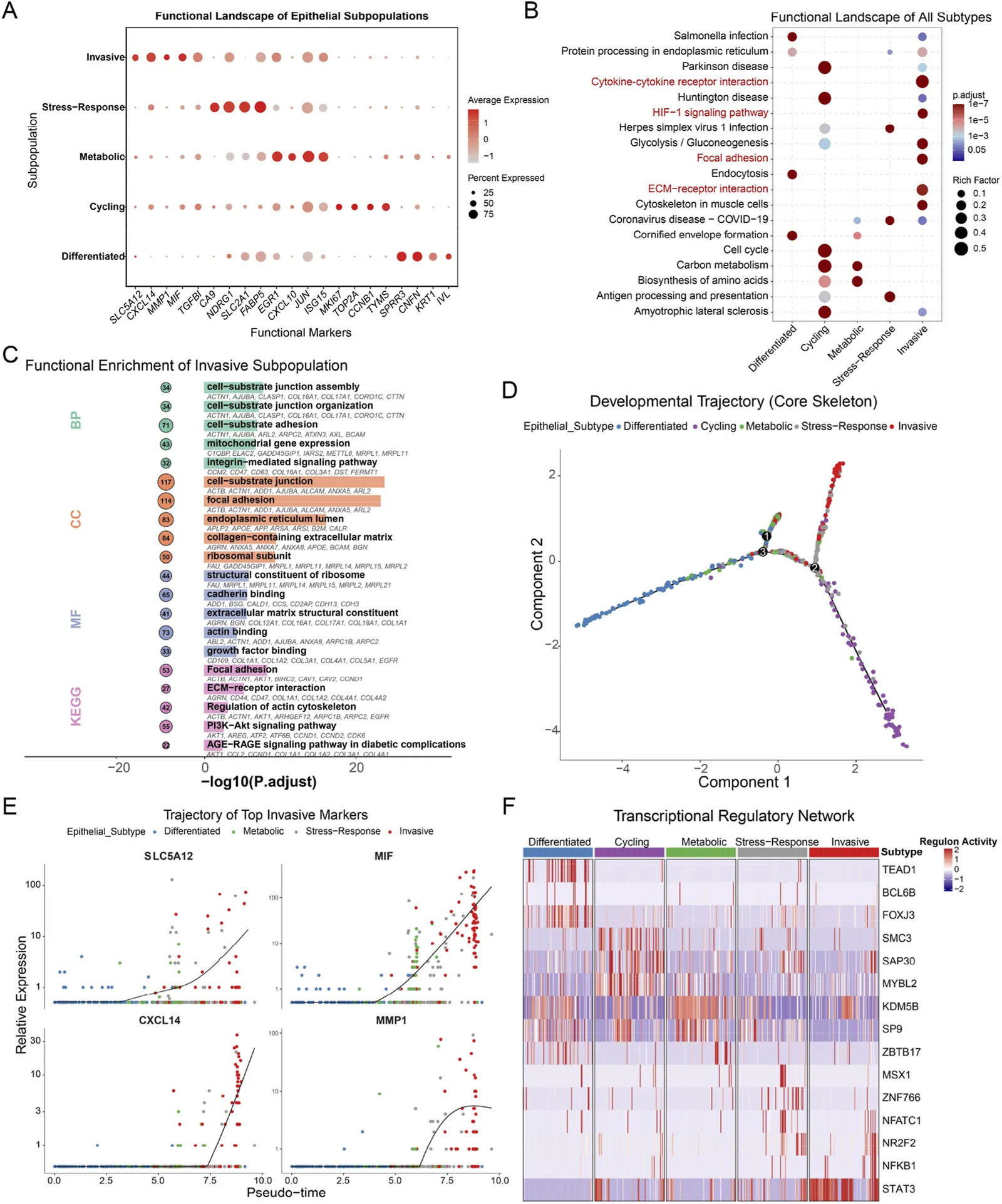
Molecular and regulatory characteristics of the Invasive epithelial state. **(A)** State-specific marker analysis highlighting increased expression of invasion-associated genes, including MMP1, CXCL14, and MIF, and reduced expression of epithelial differentiation markers such as KRT1 and IVL in the Invasive population. **(B)** KEGG enrichment of Invasive-state genes, including HIF-1 signaling, focal adhesion, and ECM-receptor interaction. **(C)** GO/pathway enrichment highlighting cell-substrate junction, extracellular-matrix, integrin, and PI3K-AKT-associated programs. **(D)** Monocle 2 trajectory reconstruction using DDRTree, showing the distribution of epithelial states along inferred pseudotime. **(E)** Pseudotime-associated expression patterns of SLC5A12, MIF, CXCL14, and MMP1, all of which increase toward the Invasive region of the trajectory. **(F)** SCENIC/AUCell regulon analysis highlighting enhanced FOSL1, STAT3, RELA, and TEAD1 activity in Invasive cells. Pseudotime reflects computational ordering rather than lineage tracing, and regulon activity does not establish direct transcriptional control.

Functional enrichment analyses reinforced this interpretation. Genes characteristic of Invasive cells were enriched in HIF-1 signaling, ECM-receptor interaction, focal adhesion, and extracellular-matrix remodeling programs (Figure 2B). Complementary pathway analyses further highlighted PI3K-AKT and integrin-related signaling, together with processes related to cell-substrate junction organization (Figure 2C). The concordance of these pathways with the marker-gene profile indicated that the Invasive state was organized around motility, adhesion, matrix interaction, and stress-adaptive programs rather than a single isolated pathway.

We then reconstructed relationships among epithelial states using Monocle 2. The inferred trajectory arranged Differentiated, Cycling, and Invasive populations along a continuous transcriptional landscape, with the Invasive state occupying a distal region relative to differentiated cells (Figure 2D). SLC5A12 increased toward this Invasive region in parallel with MIF, CXCL14, and MMP1 (Figure 2E). Because pseudotime represents computational ordering rather than lineage tracing, these data indicate that increasing SLC5A12 expression accompanies acquisition of an Invasive-like transcriptional state without establishing a direct developmental lineage.

SCENIC analysis identified a corresponding shift in regulatory activity. FOSL1, STAT3, RELA, and TEAD1 regulons were preferentially activated in Invasive cells (Figure 2F). The simultaneous enrichment of these regulons, together with ECM-, adhesion-, and invasion-related pathways, suggests that the Invasive phenotype is supported by a coordinated regulatory program rather than by a single transcriptional feature.

### 3.3 Invasive epithelial cells occupy a communication-rich niche accompanied by progressive lymphoid remodeling

Because the Invasive population combined malignant transcriptional features with strong expression of signaling molecules, we next asked whether it also occupied a distinctive position within the intercellular communication network. CellChat analysis revealed extensive ligand-receptor communication among malignant, immune, and stromal populations (Figure 3A). Fibroblasts and Invasive epithelial cells showed particularly high overall communication strength, and the Invasive population displayed prominent outgoing signaling activity (Figure 3B), indicating that this state was not only transcriptionally aggressive but also highly connected to the surrounding microenvironment.

**FIGURE 3:**
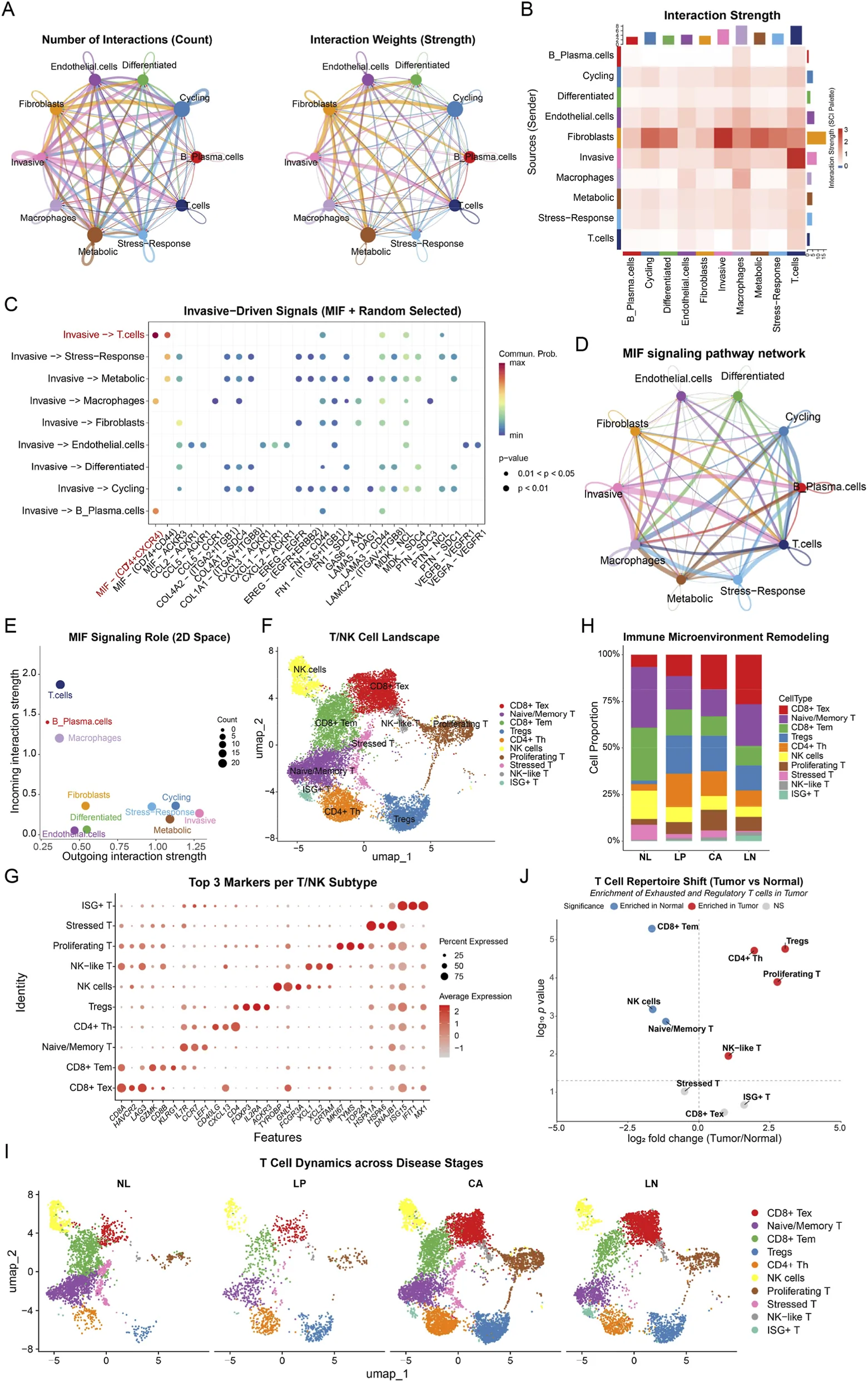
Invasive epithelial signaling is associated with remodeling of the HNSCC immune landscape. **(A)** Global CellChat network summarizing predicted intercellular communication among major cell populations. **(B)** Heatmap of predicted communication strength between sender and receiver populations. **(C)** Comparison of outgoing signaling programs across cell populations, highlighting communication activity associated with the Invasive epithelial state. **(D)** MIF-associated communication network showing predicted signaling from Invasive epithelial cells toward T cells, macrophages, and B/plasma-cell populations. **(E)** Sender/receiver analysis positioning cell populations according to their predicted signaling roles. **(F)** UMAP of T/NK-cell subpopulations after secondary clustering. **(G)** Canonical markers supporting annotation of naïve/memory T cells, cytotoxic NK cells, exhausted CD8^+^ T cells, Tregs, and other lymphoid states. **(H)** Relative frequencies of T/NK-cell states across NL, LP, CA, and LN. **(I)** Stage-resolved UMAP of T/NK-cell states. **(J)** Differential-abundance analysis showing stage-associated changes in lymphoid populations. CellChat analyses infer ligand-receptor communication from transcriptomic data and prior interaction databases and should not be interpreted as direct evidence of pathway activation.

The predicted outgoing programs from Invasive cells encompassed both extracellular-matrix and immune-associated signaling (Figures 3C–E). Collagen-related factors and FN1 contributed to predicted interactions with fibroblast and endothelial populations, consistent with the matrix-remodeling programs identified independently by pathway enrichment. In parallel, MIF emerged as a prominent immune-associated signal originating from the Invasive epithelial compartment, linking the state-level transcriptional analysis to a specific predicted tumor-immune communication axis.

To determine how these epithelial communication patterns related to the lymphoid landscape, T and NK cells were reclustered independently. Multiple immune states were resolved, including naïve/memory T cells expressing CCR7 and LEF1, cytotoxic NK cells expressing GNLY and NKG7, exhausted CD8^+^ T cells expressing HAVCR2 and LAG3, and Tregs expressing FOXP3 and CTLA4 (Figures 3F,G). This analysis established a cellular basis for evaluating stage-dependent immune remodeling beyond broad T/NK annotations.

The distribution of lymphoid states changed progressively across disease stages. Compared with NL and LP tissues, CA and LN samples contained increased proportions of exhausted CD8^+^ T cells and Tregs, whereas naïve T-cell populations were depleted (Figures 3H,I). Differential-abundance analysis further showed enrichment of Tregs, proliferating T cells, and CD4^+^ helper T cells in tumors, while NK and naïve T-cell populations were relatively more abundant in normal tissues (Figure 3J). Thus, expansion of the Invasive epithelial state occurred in an immune context increasingly enriched for exhausted and regulatory lymphocyte phenotypes.

### 3.4 MIF-CD74/CXCR4 signaling defines a preferential predicted communication route from invasive epithelial cells to Tregs

The parallel expansion of Invasive epithelial cells and Tregs prompted us to examine epithelial-to-Treg communication in greater detail. Among epithelial subpopulations, the Invasive state exhibited the strongest predicted interaction intensity with Tregs (Figures 4A,B). This pattern was accompanied by dominant outgoing activity from Invasive cells and corresponding receptor expression in Tregs, suggesting that the association was concentrated within a specific epithelial-to-immune communication route rather than reflecting uniformly elevated signaling across all epithelial states.

**FIGURE 4:**
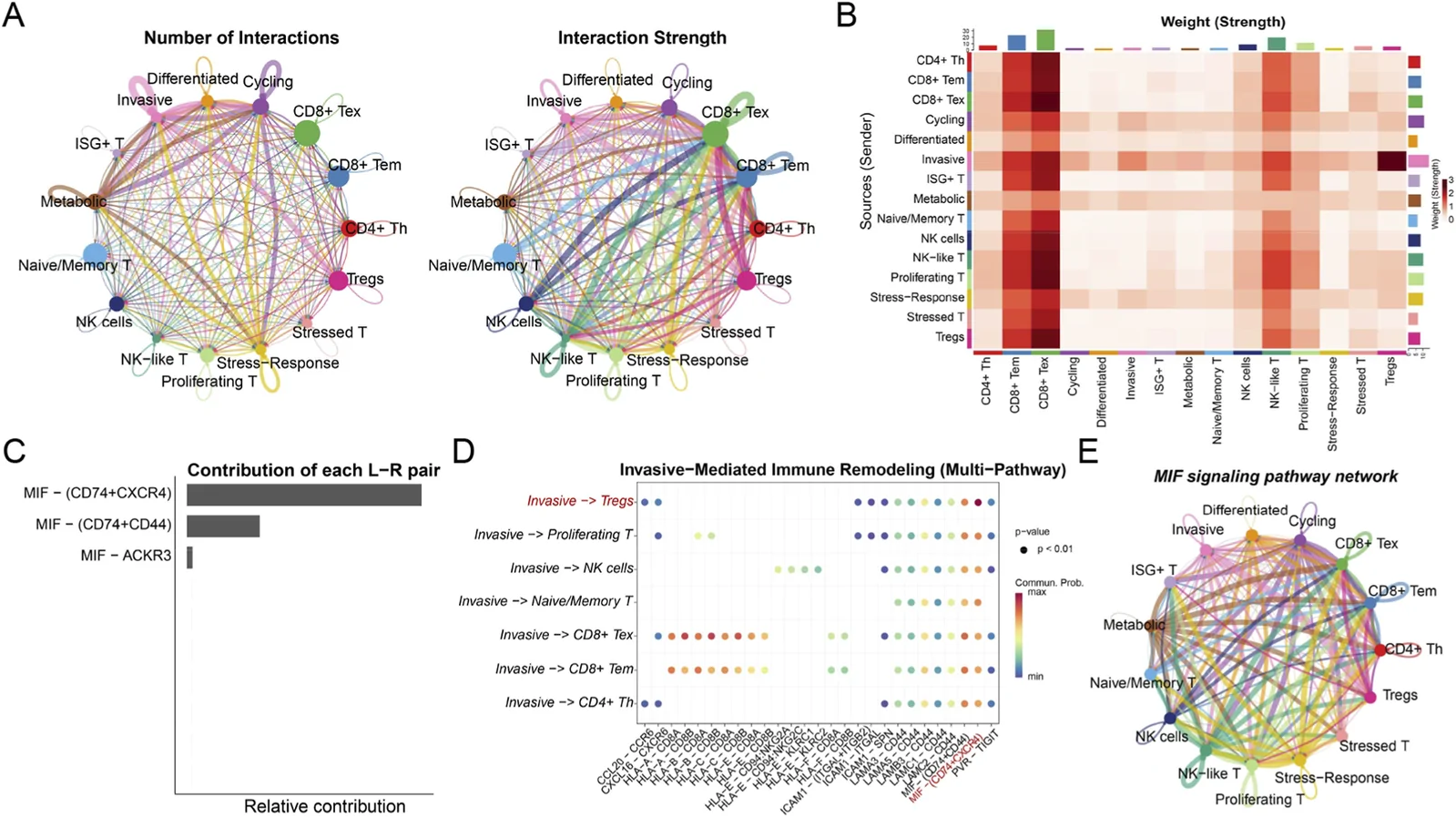
Predicted MIF-CD74/CXCR4 communication between Invasive epithelial cells and Tregs. **(A)** Circular network of predicted epithelial-to-Treg communication. **(B)** Heatmap of ligand-receptor interactions from epithelial states to Tregs, highlighting the relative prominence of MIF-associated signaling. **(C)** Relative contributions of individual ligand-receptor pairs to the predicted epithelial-to-Treg network. **(D)** Cellular distribution of MIF in Invasive epithelial cells and of CD74/CXCR4 in the Treg compartment. **(E)** Network topology of MIF-associated signaling between Invasive epithelial cells and Tregs. The MIF-CD74/CXCR4 relationship is computationally inferred and does not demonstrate ligand secretion, receptor activation, or Treg recruitment.

Ligand-receptor contribution analysis identified MIF signaling as a major component of this predicted interaction network (Figure 4C). MIF expression was elevated in Invasive epithelial cells, whereas Tregs expressed the corresponding receptor components CD74 and CXCR4 (Figure 4D). Network visualization further placed MIF-CD74/CXCR4 signaling among the prominent predicted links connecting the Invasive state to Tregs (Figure 4E).

Together, the stage-resolved abundance data and ligand-receptor inference support a model in which the Invasive epithelial state is associated with Treg-rich immune remodeling through MIF-related communication. These results remain computational: they identify a preferential signaling hypothesis but do not demonstrate direct ligand-receptor engagement, Treg recruitment, or pathway dependence.

### 3.5 Cross-cohort machine learning links the invasive-state program to patient outcome and repeatedly prioritizes SLC5A12 and MIF

We next asked whether molecular features of the single-cell-defined Invasive state could be translated into clinically informative patient-level signatures. More than 100 algorithmic configurations derived from ten survival-learning methods were evaluated in the TCGA-HNSCC cohort and across five independent GEO validation cohorts (Figure 5A). Model performance was compared using the concordance index, allowing selection of a framework on the basis of cross-cohort behavior rather than performance in a single dataset.

**FIGURE 5:**
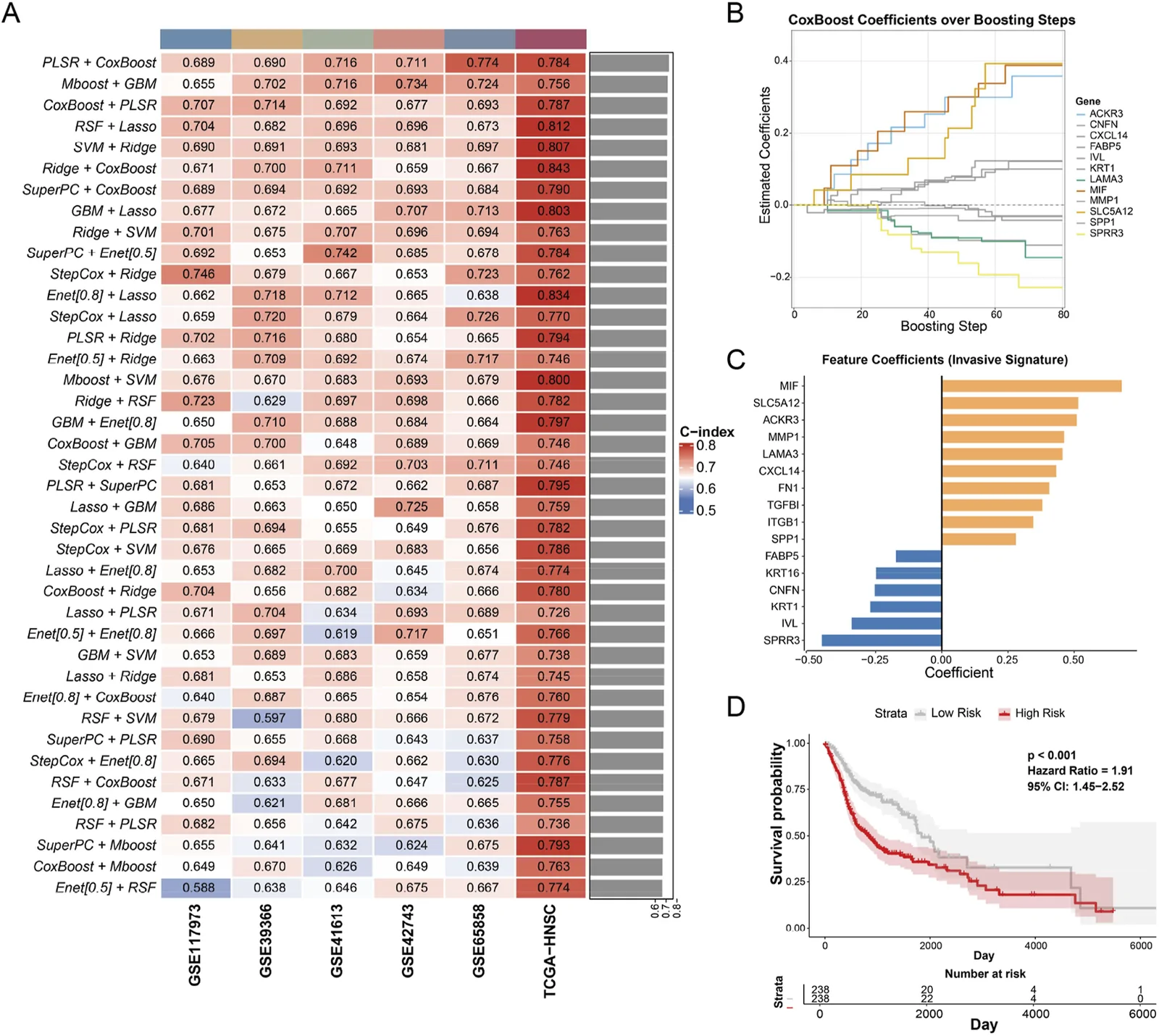
Machine-learning construction and validation of an Invasive-state-associated prognostic model. **(A)** Comparative C-index performance of the displayed top-ranked algorithmic configurations generated from the broader machine-learning framework in TCGA-HNSC and five independent GEO validation cohorts. PLSR + CoxBoost showed a balanced cross-cohort profile, with C-index values of 0.689 (GSE171973), 0.690 (GSE39366), 0.716 (GSE41613), 0.711 (GSE42743), 0.774 (GSE65858), and 0.784 (TCGA-HNSC). **(B)** CoxBoost coefficient trajectories during feature selection, including recurrent retention of MIF and SLC5A12. **(C)** Final feature coefficients, with MIF, SLC5A12, MMP1, and CXCL14 among genes with positive coefficients and differentiation-associated genes including SPRR3, IVL, and KRT1 showing coefficients in the opposite direction. **(D)** Kaplan-Meier overall-survival analysis of high- and low-risk groups (HR = 1.91, 95% CI 1.45–2.52; *P* < 0.001, log-rank test).

Across the displayed top-ranked strategies, PLSR + CoxBoost showed the most balanced cross-cohort performance (Figure 5A), with C-index values of 0.689 in GSE171973, 0.690 in GSE39366, 0.716 in GSE41613, 0.711 in GSE42743, 0.774 in GSE65858, and 0.784 in TCGA-HNSC. During feature selection, MIF and SLC5A12 were repeatedly retained (Figure 5B), indicating that both genes contributed reproducible prognostic information within the Invasive-state-associated feature space. Their recurrence was consistent with the single-cell analysis, in which both genes increased toward the Invasive region of the epithelial trajectory.

The final model contained genes with opposing prognostic contributions (Figure 5C). MIF, SLC5A12, MMP1, and CXCL14 carried positive coefficients and were associated with higher risk, whereas differentiation-related genes including SPRR3, IVL, and KRT1 carried coefficients in the opposite direction. This coefficient structure recapitulated the biological contrast observed at single-cell resolution between the Invasive program and differentiated epithelial identity.

When patients were stratified using the derived risk score, the high-risk group experienced significantly shorter overall survival than the low-risk group (HR = 1.91, 95% CI 1.45–2.52, *P* < 0.001; Figure 5D). These results demonstrate that the transcriptional architecture of the Invasive epithelial state is reflected in bulk tumors and carries clinically relevant prognostic information, while independently highlighting SLC5A12 and MIF as recurrent features of the adverse-risk program.

### 3.6 Independent transcriptomic, network, immune, spatial, and feature-selection analyses converge on SLC5A12

Given the recurrent selection of SLC5A12 in the prognostic framework, we next evaluated whether its association with the Invasive phenotype was reproducible across independent analytical layers. WGCNA of TCGA-HNSCC identified a Green co-expression module that showed the strongest positive correlations with both the Treg infiltration score (R = 0.66) and the Invasive epithelial score (R = 0.74; Figure 6A). Within this module, SLC5A12 showed high module membership and gene significance, placing it among the module features most closely aligned with the two phenotypes (Figure 6B).

**FIGURE 6:**
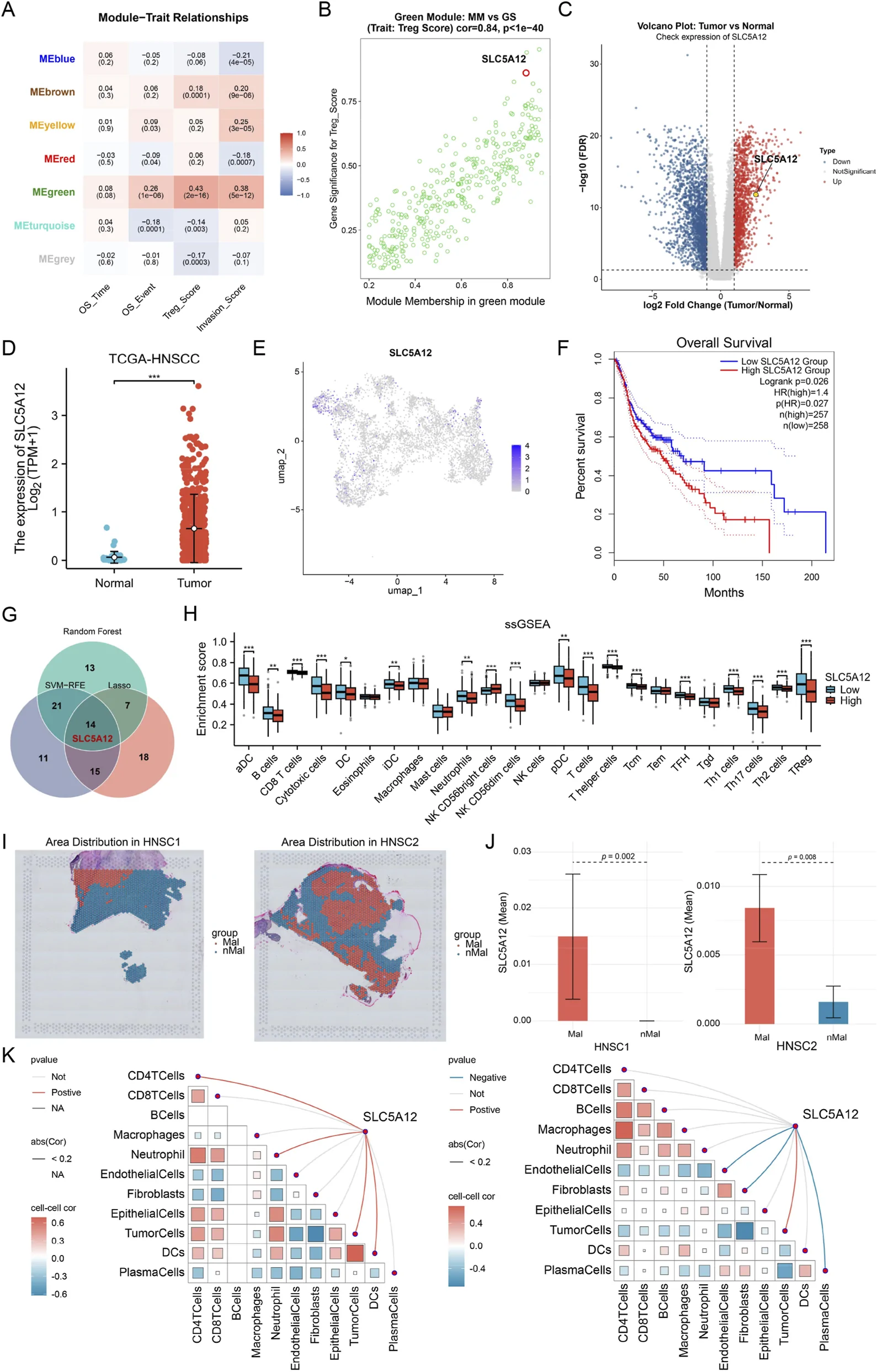
Multi-layered analyses converge on SLC5A12 as an Invasive epithelial-associated biomarker linked to immune features and clinical outcome. **(A)** WGCNA module-trait correlation heatmap. The Green module shows the strongest positive correlations with Treg infiltration score (R = 0.66) and Invasive score (R = 0.74). **(B)** Relationship between module membership and gene significance within the Green module. **(C,D)** SLC5A12 expression in HNSCC tumor and non-malignant tissues. **(E)** Single-cell localization of SLC5A12 within the Invasive epithelial state. **(F)** Overall-survival association of SLC5A12 expression. **(G)** Convergent feature selection by Random Forest, SVM-RFE, and LASSO. **(H)** Estimated immune-cell enrichment according to SLC5A12 expression/risk grouping. **(I)** Spatial distributions in GSE181300 10x Visium sections GSM5494475 (HNSCC201125T04; HNSC1) and GSM5494476 (HNSCC201125T05; HNSC2). Cell composition was inferred with Cottrazm-SpatialDecon, and spots were classified as malignant (Mal; TumorCells >0) or non-malignant (nMal; TumorCells = 0). **(J)** Comparison of SLC5A12 expression between Mal and nMal spots using the Wilcoxon rank-sum test. SLC5A12 was higher in Mal regions in both sections (HNSC1, P = 0.002; HNSC2, P = 0.008). Bars summarize group means with standard errors; spot numbers are indicated in the corresponding panels. **(K)** Spearman correlation analysis between SLC5A12 expression and Cottrazm-estimated cell-type proportions across spatial spots. **P < 0.01, ***P < 0.001.

At the tissue level, SLC5A12 expression was higher in HNSCC tumors than in non-malignant tissues and this pattern was reproduced in independent GEO datasets (Figures 6C,D). Pan-cancer analyses provided broader context by showing heterogeneous SLC5A12 expression and genomic alteration patterns across tumor types (Supplementary Figures S1,S2). At single-cell resolution, however, the signal was highly specific within HNSCC: SLC5A12 expression was preferentially concentrated in the Invasive epithelial population (Figure 6E), directly connecting the bulk-tissue observation to the malignant state identified in the scRNA-seq atlas.

Clinical analyses further supported the relevance of SLC5A12. Patients with higher SLC5A12 expression had poorer overall survival, and multivariable analysis identified SLC5A12 as an independent prognostic factor (Figure 6F). Three separate feature-selection procedures-Random Forest, SVM-RFE, and LASSO-converged on SLC5A12 as a shared candidate (Figure 6G), reducing the likelihood that its selection was specific to a single modeling strategy.

The immune and spatial analyses provided two additional levels of support. Patients with higher SLC5A12-associated risk features showed increased estimated Treg infiltration (Figure 6H), consistent with the WGCNA relationship between the Green module and Treg score. Spatial analysis was then performed in two independent GSE181300 Visium sections, HNSC1 (GSM5494475/HNSCC201125T04) and HNSC2 (GSM5494476/HNSCC201125T05; Figures 6I–K). Cottrazm-based deconvolution classified spots with a TumorCells proportion >0 as malignant (Mal) and those with TumorCells = 0 as non-malignant (nMal). SLC5A12 expression was significantly higher in Mal than nMal spots in both sections (HNSC1, P = 0.002; HNSC2, P = 0.008; Wilcoxon rank-sum test; Figure 6J). Spearman analysis across spatial spots further related SLC5A12 abundance to the Cottrazm-estimated proportions of tumor and immune/stromal cell populations (Figure 6K). These results provide an independent spatial localization layer supporting the preferential association of SLC5A12 with malignant epithelial territories, while the correlation analysis is interpreted as spatial co-variation rather than causal regulation of neighboring cell populations.

We also examined the broader transcriptional context of SLC5A12 to determine whether the main HNSCC findings were supported by independent exploratory analyses. Pan-cancer expression and copy-number analyses (Supplementary Figures S1,S2) were used to place HNSCC SLC5A12 dysregulation in a broader oncologic context, whereas pan-cancer and HNSCC immune-correlation analyses (Supplementary Figures S3,S4) assessed whether the Treg-associated pattern was part of a wider immune relationship. cMap/GDSC2 analyses (Supplementary Figures S5) were retained as hypothesis-generating pharmacogenomic context and were not used as evidence of direct SLC5A12 drug sensitivity. In TCGA-HNSC, comparison of high- and low-SLC5A12 tumors identified 668 upregulated and 233 downregulated genes at |log2FC| > 1 and adjusted P < 0.05 (Supplementary Figures S6A). KEGG/GO and GSEA analyses (Supplementary Figures S6B-I) further characterized metabolic, redox, carcinogen-response, antigen-presentation, TCR, and interferon-related programs associated with SLC5A12 expression. These supplementary analyses are therefore interpreted as supporting or exploratory context rather than as independent mechanistic proof.

### 3.7 The invasive epithelial state exhibits a coordinated ferroptosis-associated vulnerability profile

Having established SLC5A12-enriched Invasive cells as the principal biological axis of the study, we next treated ferroptosis as a downstream therapeutic-vulnerability module rather than as an experimentally validated SLC5A12 mechanism. Five complementary ferroptosis-associated module scores were compared across epithelial states. The Invasive population showed high overall ferroptosis, ferroptosis-driver, lipid-peroxidation, and iron-metabolism scores together with a low ferroptosis-suppressor score (Figures 7A,B). In contrast, Stress-Response and Differentiated cells retained comparatively stronger ferroptosis-suppressive signatures. The reciprocal behavior of pro-ferroptotic and protective programs distinguished the Invasive state at the transcriptomic level.

**FIGURE 7:**
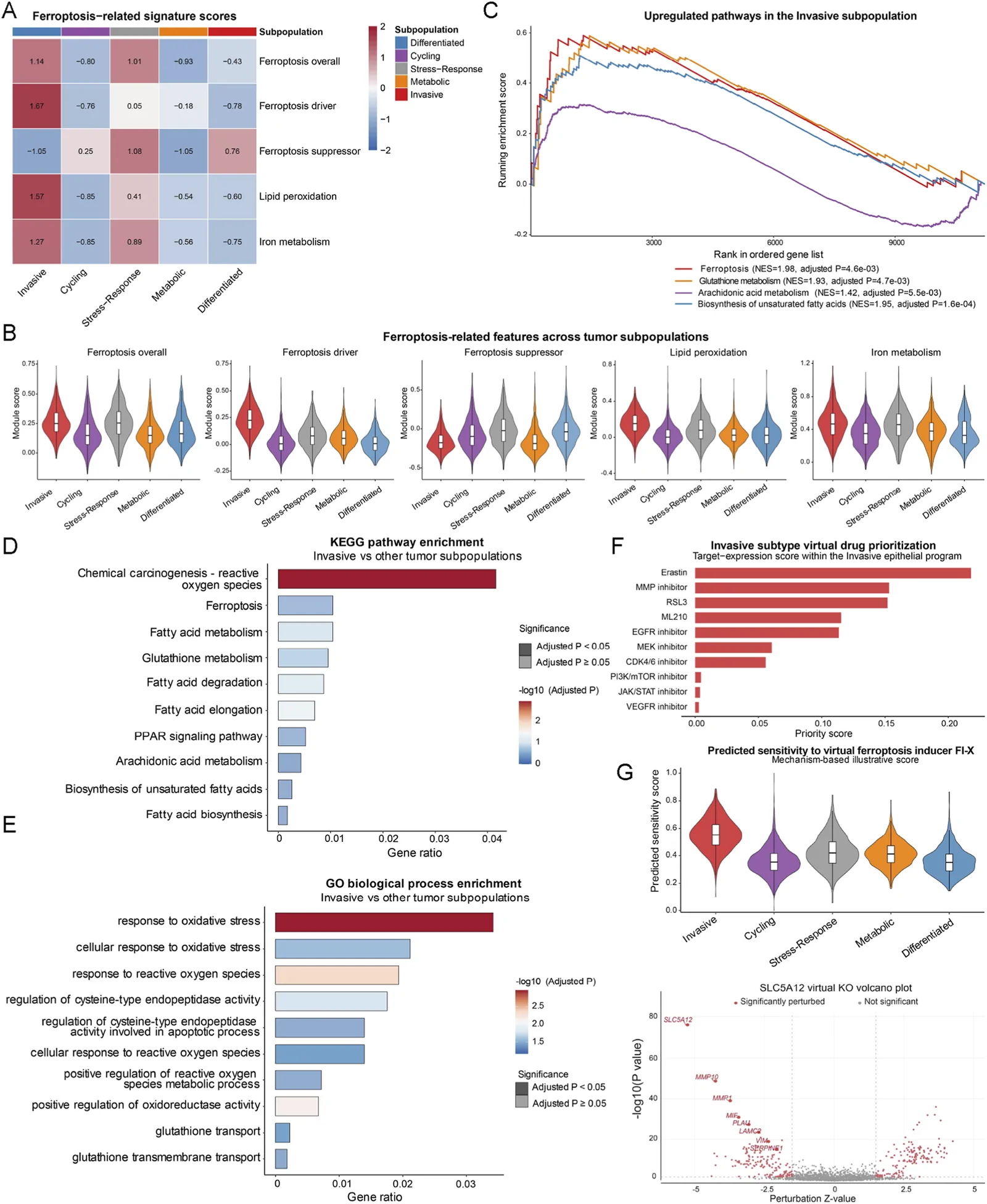
The Invasive epithelial state exhibits a ferroptosis-associated vulnerability profile and predicted sensitivity to ferroptosis-related compounds. **(A)** Heatmap of five ferroptosis-associated module scores across epithelial states: overall ferroptosis, ferroptosis driver, ferroptosis suppressor, lipid peroxidation, and iron metabolism. Scores were calculated with Seurat AddModuleScore from the predefined ten-gene regulator panel described in Methods. **(B)** Violin plots showing the single-cell distributions of the five module scores. **(C)** KEGG-based GSEA showing positive enrichment of ferroptosis, glutathione metabolism, arachidonic-acid metabolism, and biosynthesis of unsaturated fatty acids in the Invasive state. **(D)** KEGG enrichment analysis of Invasive versus other epithelial states. **(E)** GO biological-process enrichment highlighting oxidative-stress and redox-associated programs. **(F)** Beyondcell-based virtual drug prioritization in the Invasive program, with erastin ranked highest and RSL3 and ML210 also showing favorable predicted activity. **(G)** Predicted sensitivity to the hypothetical ferroptosis inducer FI-X. FI-X raw score was defined as the five-gene promoting-module score minus the five-gene protective-module score and min-max scaled to 0–1.

Pathway-level analyses independently reinforced this pattern. GSEA demonstrated positive enrichment of ferroptosis, glutathione metabolism, arachidonic-acid metabolism, and unsaturated-fatty-acid biosynthesis in Invasive cells (Figure 7C). KEGG and GO analyses further highlighted ferroptosis, reactive-oxygen-species-related processes, fatty-acid metabolism, glutathione metabolism, and oxidative-stress responses (Figures 7D,E). Thus, signature scoring and unbiased functional enrichment converged on a common redox- and lipid-metabolism-associated phenotype.

We then asked whether this transcriptional state was accompanied by predicted pharmacologic susceptibility. Virtual compound prioritization ranked erastin highest, while RSL3 and ML210 also showed high predicted activity (Figure 7F), and FI-X analysis assigned the highest predicted sensitivity to the Invasive state (Figure 7G). The agreement between signature scoring, pathway enrichment, and predicted drug sensitivity defines a computational ferroptosis-vulnerability phenotype only. No ferroptosis assay was performed, and these results do not demonstrate ferroptotic cell death or direct regulation of ferroptosis by SLC5A12.

### 3.8 Virtual SLC5A12 loss predicts coordinated reversal of invasive-state and immunoregulatory programs

Because SLC5A12 was repeatedly prioritized within the Invasive state, we next asked whether virtual perturbation of SLC5A12 was predicted to alter the transcriptional architecture of that state. CellOracle-based SLC5A12 suppression produced a coordinated predicted reduction in invasion- and mesenchymal-associated genes, including MMP10, MMP1, PLAU, LAMC2, SERPINE1, VIM, and MIF (Figure 8A), while epithelial differentiation and adhesion-associated genes, including CDH1, KRT4, CLDN4, KRT13, IVL, DSG3, and EPCAM, increased. These predictions were used to generate hypotheses for experimental phenotypic testing and were not interpreted as evidence of direct downstream regulation.

**FIGURE 8:**
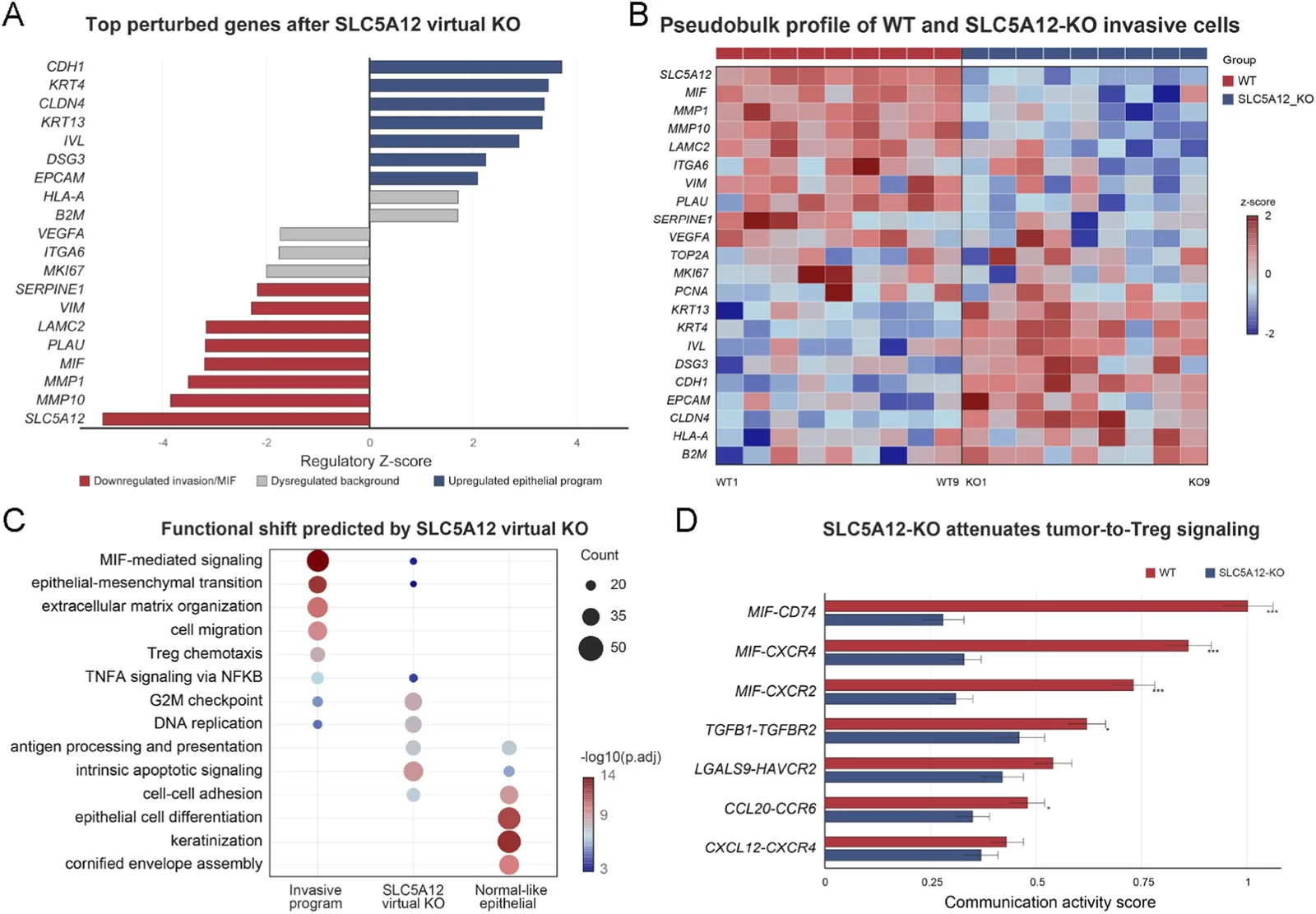
In silico SLC5A12 perturbation predicts coordinated remodeling of Invasive-state identity and immunoregulatory signaling. **(A)** Predicted gene-level response to virtual SLC5A12 suppression in Invasive epithelial cells. MMP10, MMP1, PLAU, LAMC2, SERPINE1, VIM, and MIF show reduced predicted activity, whereas CDH1, KRT4, CLDN4, KRT13, IVL, DSG3, and EPCAM show increased predicted activity. **(B)** Pseudobulk expression profiles of simulated control and SLC5A12-perturbed Invasive cells, showing coordinated reduction of invasive/mesenchymal genes and increased epithelial differentiation markers. **(C)** Pathway comparison showing lower predicted EMT, ECM remodeling, migration, MIF signaling, and Treg-chemotaxis programs after SLC5A12 perturbation, with relative enhancement of epithelial differentiation-associated programs. **(D)** Predicted changes in epithelial-to-Treg ligand-receptor communication, including reduced MIF-CD74, MIF-CXCR4, MIF-CXCR2, TGFB1-TGFBR2, and CCL20-CCR6 interactions. These analyses are *in silico* predictions and do not replace experimental genetic perturbation or pathway validation.

Pseudobulk profiles generated from simulated control and SLC5A12-perturbed Invasive cells reproduced this pattern across replicate profiles (Figure 8B). Control cells retained high expression of SLC5A12, MIF, MMP1, MMP10, LAMC2, VIM, PLAU, and SERPINE1, whereas these genes decreased after simulated SLC5A12 loss. Conversely, KRT13, KRT4, IVL, DSG3, CDH1, EPCAM, and CLDN4 increased in the perturbed state. This population-level comparison therefore supported the gene-level perturbation scores and linked SLC5A12 loss to restoration of epithelial differentiation-associated expression.

The same directional shift was evident at the pathway level. The unperturbed Invasive program was enriched for EMT, extracellular-matrix remodeling, migration, MIF signaling, and Treg chemotaxis, whereas these programs showed lower predicted activity following SLC5A12 perturbation; epithelial differentiation-related programs increased in parallel (Figure 8C). These results connected the gene-level response to higher-order processes that had independently characterized the Invasive state in Figures 1–4.

Finally, ligand-receptor analysis predicted that virtual SLC5A12 suppression would weaken MIF-CD74, MIF-CXCR4, MIF-CXCR2, TGFB1-TGFBR2, and CCL20-CCR6 communication (Figure 8D). This concordance links SLC5A12 expression computationally to Invasive identity and immunoregulatory signaling but does not show that SLC5A12 directly controls MIF signaling or Treg recruitment. We therefore moved from prediction to direct testing of SLC5A12 itself, focusing on experimentally measurable HNSCC cell and xenograft phenotypes rather than on the predicted immune pathway.

### 3.9 Genetic depletion of SLC5A12 suppresses HNSCC cell growth and increases apoptotic cell death

To experimentally test the functional relevance of SLC5A12, we established stable SLC5A12-depleted FaDu and CAL27 cell models using two independent shRNAs. Immunoblotting confirmed effective reduction of SLC5A12 protein in both cell lines relative to the sh-NC controls (Figure 9A), providing two independent loss-of-function conditions for subsequent phenotypic analyses.

**FIGURE 9:**
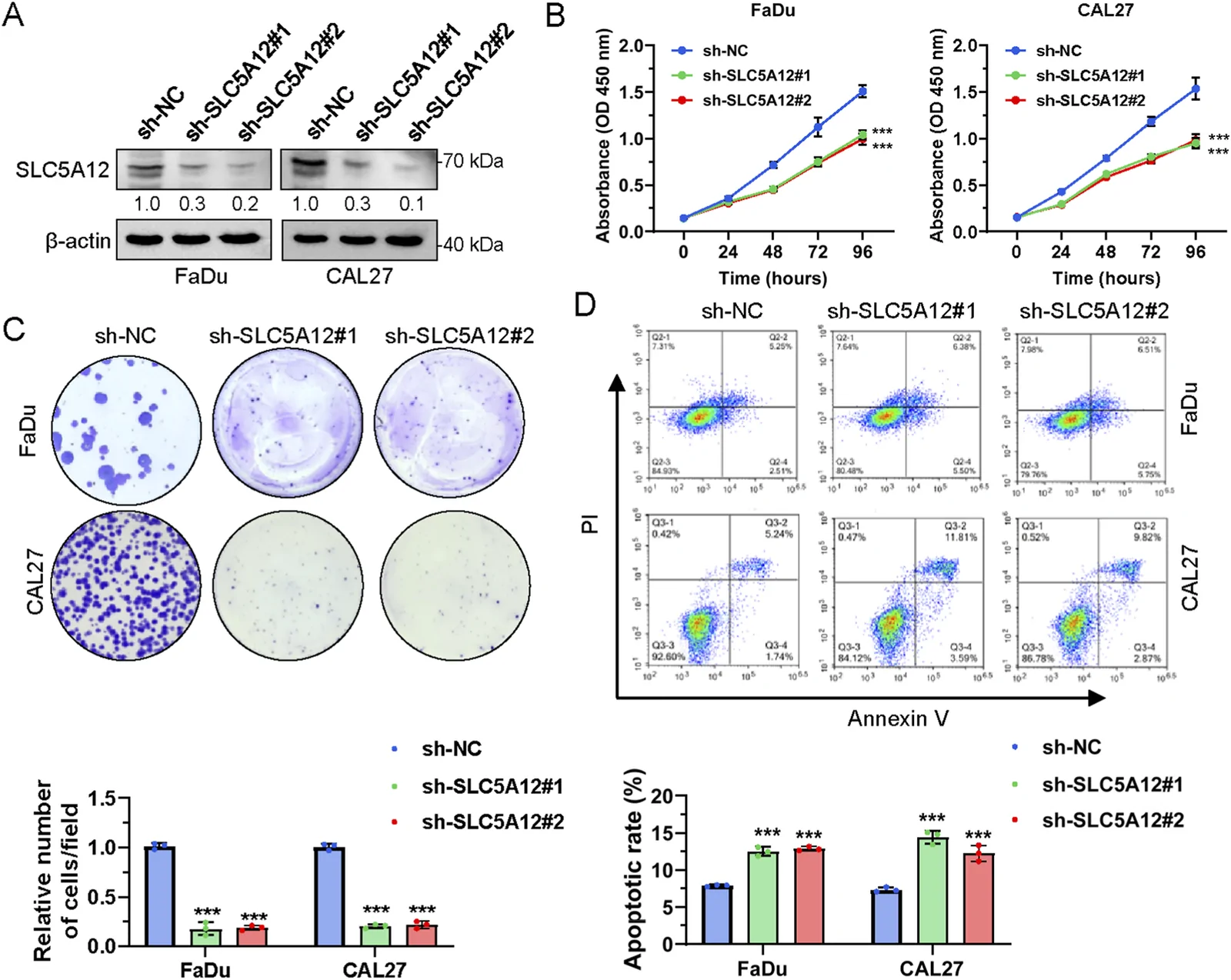
SLC5A12 depletion restricts HNSCC cell growth and increases apoptotic cell death *in vitro*. **(A)** Representative Western blots confirming SLC5A12 knockdown in FaDu and CAL27 cells expressing sh-NC, sh-SLC5A12#1, or sh-SLC5A12#2; beta-actin was used as the loading control. **(B)** CCK-8 growth curves measured at the indicated time points, showing reduced cell accumulation after SLC5A12 depletion. **(C)** Representative colony-formation images and quantification after 14 days, demonstrating reduced clonogenic capacity in SLC5A12-silenced cells. **(D)** Representative flow-cytometric apoptosis plots and corresponding quantification showing increased apoptosis after SLC5A12 knockdown. Colony formation and apoptosis results are derived from three independent biological experiments; CCK-8 measurements were performed with technical replicates as described in Methods. Data are presented as mean ± SD where applicable. Statistical significance is shown in the corresponding panels. ***P < 0.001.

CCK-8 assays showed that SLC5A12 depletion consistently reduced cell growth in FaDu and CAL27 cells, with both shRNAs producing lower proliferation trajectories than the corresponding controls (Figure 9B). The effect was reproduced in colony-formation assays: SLC5A12-silenced cells generated markedly fewer colonies, indicating impaired long-term clonogenic capacity (Figure 9C). The agreement between short-term growth and clonogenic assays demonstrated that the phenotype was not restricted to a single experimental readout.

Flow-cytometric analysis further showed an increased apoptotic fraction after SLC5A12 knockdown in both HNSCC models (Figure 9D). Thus, the reduction in cell accumulation was accompanied by increased apoptotic cell death. These experiments establish a functional requirement for SLC5A12 in maintaining HNSCC cell growth and survival under the conditions tested. The apoptosis data are distinct from the computational ferroptosis analysis and are not interpreted as experimental validation of ferroptosis.

### 3.10 SLC5A12 depletion impairs the migratory and invasive capacities of HNSCC cells

Because SLC5A12 was preferentially enriched in the single-cell-defined Invasive state, we next determined whether its depletion altered cell motility. Transwell migration assays demonstrated a marked reduction in the number of migrating FaDu cells following SLC5A12 knockdown, and the same phenotype was observed in CAL27 cells (Figures 10A–C). The consistency across two independent HNSCC cell lines paralleled the growth phenotype observed in Figure 9.

**FIGURE 10:**
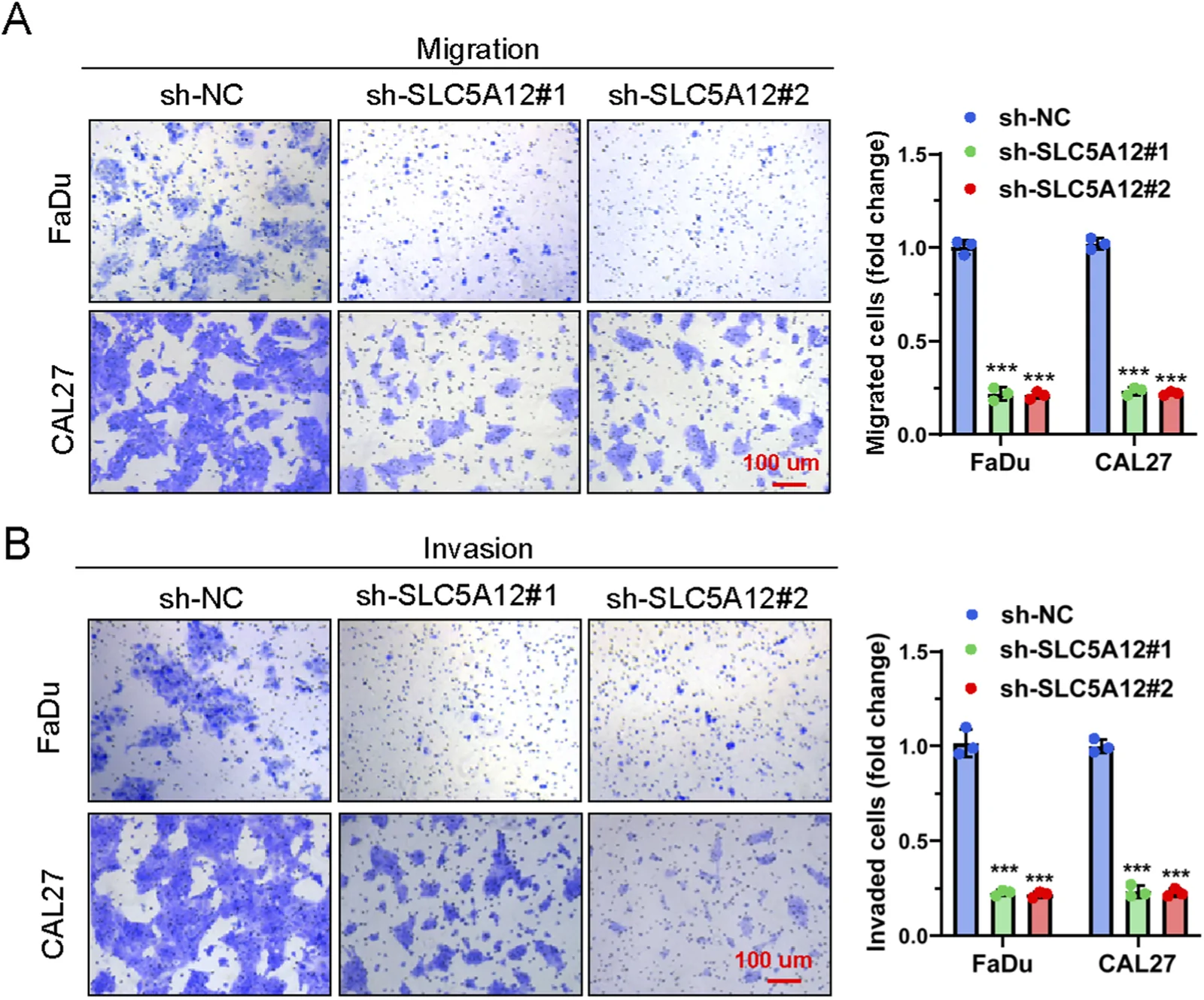
SLC5A12 depletion compromises migration and invasion in HNSCC cells. **(A)** Representative Transwell migration images and quantification for FaDu cells following SLC5A12 knockdown. **(B)** Representative Matrigel-coated Transwell invasion images and quantification for FaDu cells. **(C)** Representative Transwell migration images and quantification for CAL27 cells. **(D)** Representative Matrigel invasion images and quantification for CAL27 cells. For each insert, migrated or invaded cells were quantified from randomly selected microscopic fields as described in Methods. Experiments were independently repeated in triplicate. Data are presented as mean ± SD where applicable, and statistical significance is shown in the corresponding panels. ***P < 0.001.

Matrigel-coated Transwell assays showed an analogous effect on invasion. SLC5A12 depletion significantly reduced the invasive capacity of both FaDu and CAL27 cells relative to their respective sh-NC controls (Figures 10B–D). Thus, SLC5A12 loss affected both uncoated migration and matrix-dependent invasion, providing experimental support for the association between SLC5A12 expression and the Invasive epithelial program identified by single-cell analysis.

### 3.11 SLC5A12 depletion restrains HNSCC xenograft growth *in vivo*

We next evaluated whether the growth-suppressive effect of SLC5A12 depletion was maintained *in vivo*. FaDu cells carrying sh-SLC5A12#2 or sh-NC were implanted subcutaneously into the bilateral axillary regions of female BALB/c nude mice. Tumor growth was monitored from day 7, and all animals were included in the final analysis (n = 5 mice per group; Figures 11A,B). Each mouse was treated as the independent biological unit, with measurements from the bilateral tumors averaged for statistical analysis.

**FIGURE 11:**
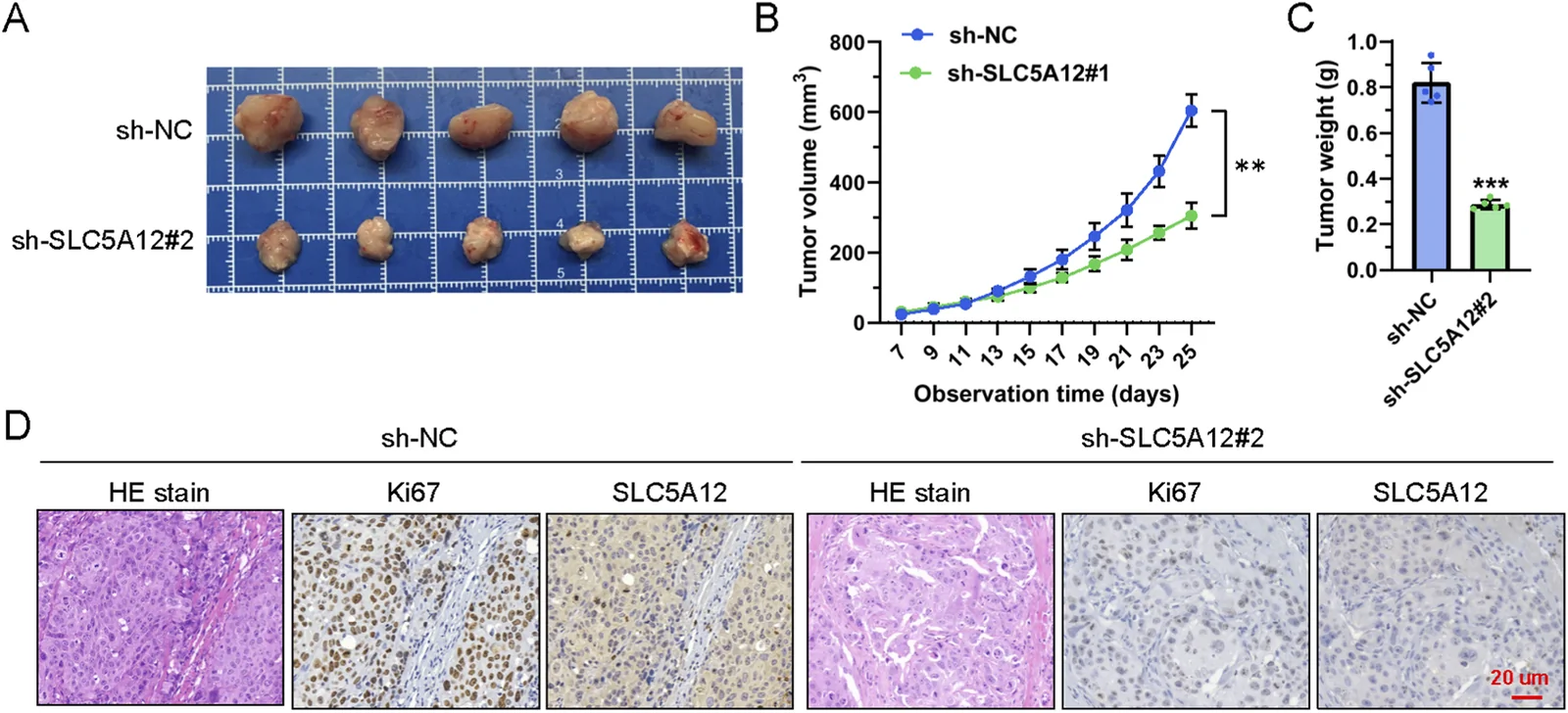
SLC5A12 depletion restrains HNSCC xenograft growth *in vivo*. **(A)** Representative tumors harvested on day 25 from female BALB/c nude mice implanted bilaterally with sh-NC or sh-SLC5A12#2 FaDu cells. **(B)** Longitudinal tumor-growth curves measured every 2 days beginning on day 7 after inoculation. Tumor volume was calculated as length x width^2/2. **(C)** Endpoint tumor weights on day 25. **(D)** Representative immunohistochemical staining for SLC5A12 and Ki67 in xenograft tissues, showing lower staining in the SLC5A12-knockdown group. n = 5 mice per group. The mouse was treated as the independent biological unit, and bilateral tumor measurements were averaged before statistical analysis. No animals were excluded from the final analysis. Statistical significance is shown in the corresponding panels. **P < 0.01, ***P < 0.001.

Tumors derived from SLC5A12-depleted cells displayed slower growth kinetics than control xenografts throughout follow-up (Figure 11B). At the day-25 endpoint, the SLC5A12-knockdown group showed lower tumor burden, including reduced final tumor weights (Figures 11A,C). These *in vivo* findings were directionally consistent with the reduced proliferation and clonogenic capacity observed in cultured cells.

Immunohistochemical analysis provided tissue-level confirmation of the experimental phenotype. Xenografts generated from SLC5A12-depleted cells showed lower SLC5A12 staining and reduced Ki67 labeling compared with control tumors (Figure 11D), consistent with effective target depletion and reduced proliferative activity *in vivo*. No animals were excluded and no adverse events were reported during the experiment. Together, the cell-based and xenograft data establish that SLC5A12 contributes to HNSCC growth across complementary experimental systems.

### 3.12 Virtual screening and molecular docking nominate two candidate compounds with favorable predicted interactions with SLC5A12

Having established a functional role for SLC5A12, we next used structure-based virtual screening to identify compounds with favorable predicted interactions with the protein. FDA-approved compounds retrieved from the ZINC database were initially screened against the AlphaFold-derived SLC5A12 structure. The ten compounds with the lowest predicted docking energies were retained for refined analysis, revealing a range of predicted affinities across the top-ranked candidates (Figure 12A).

**FIGURE 12:**
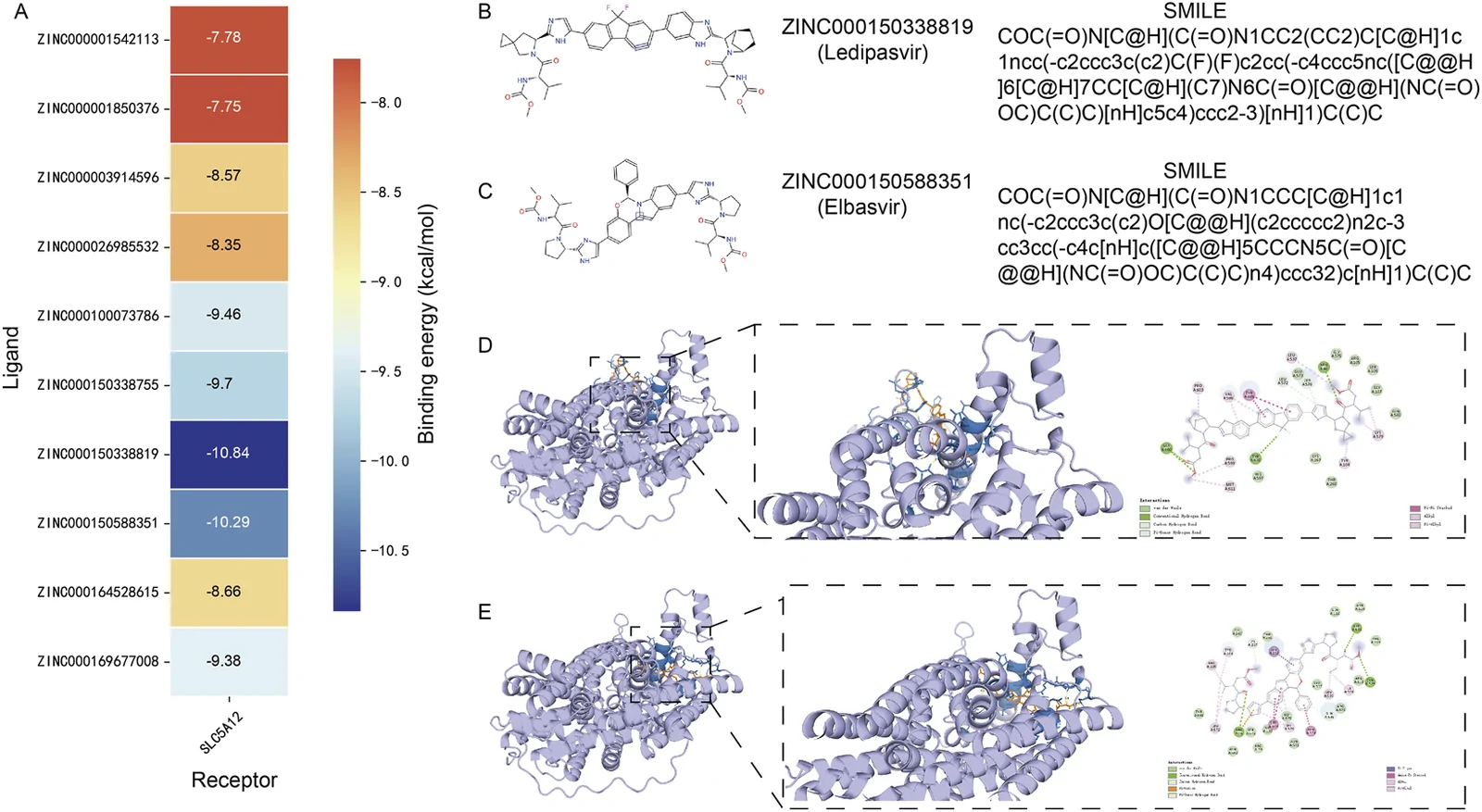
Virtual screening and molecular docking identify candidate compounds with favorable predicted interactions with SLC5A12. **(A)** Heatmap of predicted AutoDock Vina docking energies (kcal/mol) for the ten highest-ranked FDA-approved compounds screened against the AlphaFold-derived SLC5A12 structure. More negative values indicate more favorable predicted docking. Ledipasvir (ZINC000150338819) and elbasvir (ZINC000150588351) were selected for detailed visualization. Refined docking used a search box centered at (2.031, 4.912, −11.067) with dimensions (29.522, 24.504, 37.324), exhaustiveness = 32, seed = 42, num_modes = 10, and energy_range = 3 kcal/mol. **(B)** Chemical structure and SMILES of ledipasvir **(C)** Chemical structure and SMILES of elbasvir. **(D)** Lowest-energy pose and interaction map for ledipasvir-SLC5A12, showing nine predicted hydrogen bonds and four hydrophobic contacts involving ARG46, GLY107, PRO599, GLY600, and MET611. **(E)** Lowest-energy pose and interaction map for elbasvir-SLC5A12, showing six predicted hydrogen bonds and seven hydrophobic contacts involving ARG46, GLN529, ASP533, ILE534, and GLN535. Docking energies are computational ranking scores and do not establish physical binding or inhibitory activity.

Ledipasvir (ZINC000150338819) and elbasvir (ZINC000150588351) produced the most favorable docking results among the screened compounds and were therefore selected for detailed structural visualization (Figures 12B,C). Inspection of the lowest-energy poses revealed distinct predicted interaction networks within the modeled SLC5A12 binding environment.

The ZINC000150338819-SLC5A12 complex contained nine predicted hydrogen bonds and four hydrophobic contacts. ARG46, GLY107, GLY600, MET611, and PRO599 were among the key residues involved in the modeled interaction (Figure 12D). By comparison, the ZINC000150588351-SLC5A12 complex contained six predicted hydrogen bonds and seven hydrophobic contacts, with ARG46, ASP533, GLN529, GLN535, and ILE534 contributing to the interaction network (Figure 12E). ARG46 was therefore represented among the modeled interacting residues of both top-ranked complexes.

These docking results provide a structural basis for prioritizing ZINC000150338819 and ZINC000150588351 for subsequent experimental assessment. Because the present analysis is entirely computational, the compounds are described as candidate SLC5A12-binding molecules rather than validated inhibitors, and the docking scores do not establish physical binding or antitumor activity.

## 4 Discussion

This study resolves an SLC5A12-enriched Invasive epithelial state as the principal biological axis linking HNSCC progression, immune-associated communication, prognosis, and predicted therapeutic vulnerability. Single-cell studies have likewise shown that HNSCC malignant epithelial cells comprise functionally distinct transcriptional states associated with progression and treatment resistance (Liu et al., 2022; Lin et al., 2022; Zhang et al., 2024; Mohamed et al., 2026; Wang et al., 2024). Our contribution is the convergence of state-level localization, patient-level prognosis, spatial enrichment, computational perturbation, and experimental SLC5A12 loss-of-function evidence within the same analytical framework, rather than the identification of epithelial heterogeneity alone.

The stage-resolved distribution and pseudotime analyses place the Invasive state within a broader continuum of epithelial remodeling. Previous studies have described ecosystem evolution from leukoplakia to HNSCC, differences between primary and lymph-node microenvironments, immune-modulating malignant programs, MMP1-associated immune escape, and stem-like epithelial populations in head and neck cancer (Zhang et al., 2024; Wang et al., 2024; Wang et al., 2025; Fang et al., 2024; Chen et al., 2025). In the present study, SLC5A12, MIF, CXCL14, and MMP1 increased toward the Invasive region while differentiation markers declined. Because pseudotime is computational ordering, this pattern is interpreted as state association rather than a fixed developmental lineage.

The Invasive state was also associated with increased FOSL1, STAT3, RELA, and TEAD1 regulon activity. AP-1- and NF-kB-related programs have been linked to metastatic plasticity and immune suppression in other cancers (Rong et al., 2022; Zhang et al., 2022; Chen et al., 2021; Ogden et al., 2025; Chou et al., 2024), whereas STAT3 and RELA have direct relevance to HNSCC stemness, PD-1/PD-L1-associated immunosuppression, oral squamous cell carcinoma progression, and treatment resistance (Johnson et al., 2020; Li J. et al., 2024; Bu et al., 2017). These precedents provide biological context for the co-enrichment observed here, but our SCENIC analysis does not establish direct transcriptional control of SLC5A12 or MIF by these factors.

A second dimension of the Invasive state is its predicted relationship with the immune microenvironment. CellChat identified MIF-associated signaling, particularly MIF-CD74/CXCR4, as a prominent Invasive-to-Treg route. MIF is a pleiotropic inflammatory mediator with established immune-regulatory functions (Sumaiya et al., 2022; Bozza et al., 2020; Huang et al., 2025), and cancer-associated work further illustrates its capacity to participate in systemic tumor phenotypes (Li J. et al., 2024; Yang et al., 2023; Hu et al., 2022). In our data, MIF was enriched in Invasive epithelial cells and CD74/CXCR4 were present in Tregs. This supports a biologically plausible communication hypothesis, but not direct receptor activation, Treg recruitment, or pathway dependence.

The same Invasive state was enriched for extracellular-matrix and adhesion programs. Integrative single-cell/spatial analyses in other tumors have identified ECM-remodeling stromal programs associated with cancer progression, and the reciprocal interaction between matrix remodeling and cancer therapy is well established (Hu et al., 2022; Cui et al., 2025). In HNSCC, this co-occurrence suggests that the Invasive state sits at the intersection of tumor-cell motility, matrix interaction, and immune-associated signaling, without requiring that any one of these programs be causally upstream of the others.

SLC5A12 emerged from this framework as a reproducible state-associated molecule rather than from a single screening step. Its enrichment in Invasive cells, increase along the Invasive-directed pseudotime region, high WGCNA module membership/gene significance, adverse survival association, spatial localization, and repeated selection by independent feature-selection procedures provide orthogonal evidence. Prior studies link SLC5A12 to lactate-dependent inflammatory signaling and tumor-associated metabolic phenotypes (Certo et al., 2025; Lin et al., 2025), while machine-learning approaches have been used to prioritize immune-related genes in head and neck cancer (Qin et al., 2025). Most importantly, our genetic depletion experiments show that SLC5A12 contributes to HNSCC growth, clonogenicity, migration, invasion, and xenograft expansion.

The CellOracle results extend this association by predicting that SLC5A12 loss shifts Invasive cells away from mesenchymal/invasive programs and reduces MIF-, TGFB1-, and CCL20-associated communication. The predicted reduction in Treg-related signaling is directionally consistent with evidence that MIF can influence Th17/Treg balance in inflammatory disease (Zhang et al., 2025). However, this comparison is hypothesis-generating only: the current data do not establish that SLC5A12 directly regulates MIF transcription, MIF receptor signaling, or Treg recruitment.

The ferroptosis analysis represents a distinct downstream vulnerability module rather than a mechanistic extension of the SLC5A12 knockdown phenotype. Ferroptosis has been implicated in HNSCC experimentally and through single-cell analyses (Shi et al., 2026; Liu et al., 2022). Here, Invasive cells showed higher ferroptosis-promoting, lipid-peroxidation, and iron-metabolism scores, lower ferroptosis-suppression scores, and higher predicted sensitivity to ferroptosis-related compounds. By contrast, the experimental flow-cytometry data demonstrate increased apoptosis after SLC5A12 depletion. These are separate observations: apoptosis provides experimental evidence of altered cell survival, whereas ferroptosis remains a computationally predicted vulnerability and is not used to infer a shared cell-death mechanism.

The structure-based screen similarly serves as a therapeutic hypothesis-generating module. Ledipasvir and elbasvir were the top-ranked candidates in the submitted docking analysis and formed favorable predicted interactions with the AlphaFold-derived SLC5A12 structure. Docking alone does not establish biochemical binding, transporter inhibition, target engagement, or antitumor efficacy. Accordingly, these compounds are presented as candidates for future testing rather than as validated SLC5A12 inhibitors.

Several limitations define the evidence boundaries of the study. Public scRNA-seq and spatial datasets differ in sampling and technical composition; ligand-receptor communication and CellOracle perturbation are computational predictions; ferroptosis was not directly measured; SLC5A12 functional validation is predominantly loss-of-function based; and the docking candidates remain untested experimentally. These limitations are stated explicitly so that association, computational prediction, and experimentally supported function are not conflated.

Taken together, the novelty of this work lies in the convergence of four evidence layers around a single biological axis: (i) an Invasive epithelial state that expands with HNSCC progression, (ii) enrichment of SLC5A12 within that state and association with adverse outcome, (iii) predicted MIF/Treg-associated communication and ferroptosis vulnerability, and (iv) direct functional evidence that SLC5A12 depletion suppresses malignant HNSCC phenotypes. This organization also places the ferroptosis and docking analyses in their appropriate role as downstream therapeutic hypotheses rather than experimentally established mechanisms.

## 5 Conclusion

In summary, this study identifies an SLC5A12-enriched Invasive epithelial state that expands during HNSCC progression and is associated with inferred CNV burden, malignant transcriptional programs, adverse outcome, and predicted MIF-CD74/CXCR4 communication with Tregs. SLC5A12 depletion experimentally suppresses HNSCC proliferation, clonogenicity, migration, invasion, and xenograft growth while increasing apoptosis. In parallel, the Invasive state displays a computational ferroptosis-associated vulnerability profile, and virtual screening nominates ledipasvir and elbasvir as candidate SLC5A12-binding molecules. The immune-communication, ferroptosis, CellOracle, and docking results remain computational predictions; the experimentally supported conclusion is that SLC5A12 contributes to malignant HNSCC phenotypes and represents a candidate target for further mechanistic and pharmacologic investigation.

## Data Availability

The original contributions presented in the study are included in the article/Supplementary Material, further inquiries can be directed to the corresponding author.
