# Supplementary material for: SLC5A12 defines an invasive epithelial state associated with immune remodeling and therapeutic vulnerability in HNSCC
Source: Front Cell Dev Biol. 2026 Oct 22;14:1991399. doi: 10.3389/fcell.2026.1991399 (PMC13736759; doi:10.3389/fcell.2026.1991399)
Supplement: Supplementary file 1 [file DataSheet1.docx]

**SLC5A12 Defines an Invasive Epithelial State Associated with Immune Remodeling and Therapeutic Vulnerability in HNSCC**

**Supplementary Figure 1. Pan-cancer transcriptional landscape of SLC5A12.**

(A) Distribution of SLC5A12 transcript abundance across cancer types represented in The Cancer Genome Atlas (TCGA). (B) Pan-cancer comparison of SLC5A12 expression between malignant and non-malignant tissues using integrated TCGA and Genotype-Tissue Expression (GTEx) data. (C) Tumor-versus-normal comparison of SLC5A12 transcript levels across individual cancer types, using matched or corresponding non-malignant tissues where available. ns *P* >0.05, * *P* <0.05, ** *P* <0.01, *** *P* <0.001.

**Supplementary Figure S2. Pan-cancer genomic alteration profile of SLC5A12.**

**(A)** Spectrum of SLC5A12 genomic alterations across cancer types, showing the distribution of different alteration categories within pan-cancer cohorts. **(B)** Frequency and distribution of copy-number alterations (CNAs) affecting the SLC5A12 locus across individual malignancies. **(C)** Relationship between SLC5A12 copy-number status and transcript abundance across tumor types. Correlation analysis evaluates the association between genomic dosage alterations and corresponding SLC5A12 mRNA levels.

**Supplementary Figure S3. Pan-cancer associations between SLC5A12 expression and immune-cell infiltration.**

Correlation landscape depicting the relationships between SLC5A12 transcript abundance and estimated infiltration levels of immune-cell populations across tumor types in The Cancer Genome Atlas (TCGA). Correlation direction and magnitude are represented by the corresponding color scale, with statistical significance indicated as defined in the figure. These patterns characterize cancer-type-dependent associations between SLC5A12 expression and the immune microenvironment. * *P* <0.05, ** *P* <0.01, *** *P* <0.001.

**Supplementary Figure S4. Associations between SLC5A12 expression and immune-cell signature enrichment in HNSCC.**

(A-H) Pearson correlation analyses examining the relationships between SLC5A12 transcript abundance and estimated enrichment scores for eight immune-cell signatures: activated dendritic cells (aDCs) (A), B cells (B), cytotoxic cells (C), CD56 dim natural killer cells (D), T cells (E), T helper 1 (Th1) cells (F), T helper 17 (Th17) cells (G), and regulatory T cells (Tregs) (H). Correlation coefficients and corresponding statistical significance are displayed in each panel.

**Supplementary Figure S5. Computational identification of SLC5A12-associated candidate compounds and pharmacogenomic response patterns.**

(A) Connectivity Map (cMap)-based prioritization of candidate compounds associated with SLC5A12-related transcriptional signatures across cancer types. The heatmap summarizes compound rankings according to XSum scores, highlighting agents predicted to perturb or reverse SLC5A12-associated expression programs. (B) Pharmacogenomic analysis integrating SLC5A12 expression with drug-response profiles from the Genomics of Drug Sensitivity in Cancer 2 (GDSC2) resource. The top 30 compounds showing the strongest significant associations with SLC5A12 transcript abundance are displayed, with correlation direction and magnitude indicating the relationship between SLC5A12 expression and predicted drug-response patterns.

**Supplementary Figure S6. SLC5A12-associated transcriptional alterations and pathway enrichment patterns in HNSCC.**

(A) Differential transcriptomic analysis between HNSCC samples with high and low SLC5A12 expression. The volcano plot displays genes showing significant expression differences between the two groups, stratified according to the median SLC5A12 transcript level. (B-I) Functional characterization of SLC5A12-associated transcriptional patterns using Kyoto Encyclopedia of Genes and Genomes (KEGG), Gene Ontology (GO), and Gene Set Enrichment Analysis (GSEA). These analyses identify biological programs associated with differences in SLC5A12 expression, including metabolic, redox-related, carcinogen-response, and immune-associated pathways. GSEA further characterizes pathways showing positive or negative enrichment in relation to SLC5A12 abundance.
